# Aqueous Two‐Phase Enabled Low Viscosity 3D (LoV3D) Bioprinting of Living Matter

**DOI:** 10.1002/advs.202204609

**Published:** 2022-12-30

**Authors:** Malin Becker, Melvin Gurian, Maik Schot, Jeroen Leijten

**Affiliations:** ^1^ Leijten Lab Dept. of Developmental BioEngineering TechMed Centre University of Twente Enschede 7522 NB The Netherlands

**Keywords:** biofabrication, biofunctionalization, embedded bioprinting, tissue engineering, vascularization

## Abstract

Embedded 3D bioprinting has great value for the freeform fabrication of living matter. However, embedded 3D bioprinting is currently limited to highly viscous liquid baths or liquid‐like solid baths. In contrast, prior to crosslinking, most hydrogels are formulated as low‐viscosity solutions and are therefore not directly compatible with bioprinting due to low shape fidelity and poor print stability. The authors here present a method to enable low‐viscosity ink 3D (LoV3D) bioprinting, based on aqueous two‐phase stabilization of the ink‐bath interface. LoV3D allows for the printing of living constructs at high extrusion speeds (up to 1.8 m s^−1^) with high viability due to its exceedingly low‐viscosity. Moreover, LoV3D liquid/liquid interfaces offer unique advantages for fusing printed structures, creating intricate vasculature, and modifying surfaces at higher efficiencies than traditional systems. Furthermore, the low interfacial tension of LoV3D bioprinting offers unprecedented nozzle‐independent control over filament diameter via large‐dimension strand‐thinning, which allows for the printing of an exceptionally wide range of diameters down to the width of a single cell. Overall, LoV3D bioprinting is a unique all‐aqueous approach with broad material compatibility without the need for rheological ink adaption, which opens new avenues of application in cell patterning, drug screening, engineered meat, and organ fabrication.

## Graphical abstract

A facile method to enable low‐viscosity 3D (LoV3D) printing based on aqueous two‐phase stabilization is proposed, which offers accurate print‐strand thinning down to single‐cell resolution. LoV3D allows for extremely fast bioprinting at high cell viability, enables the printing of smooth, intricate, fused canicular networks, and improves the efficiency of surface functionalization, making it a promising tool for numerous bioengineering applications.

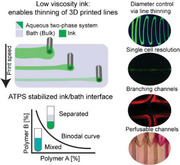

## 1 Introduction

3D (bio)printing has emerged as a major driving force enabling the creation of viable, architected, clinically‐sized engineered tissues.^[^ ^1^ ^]^ In the last decade, various printing techniques have been developed including stereolithography, drop‐on‐demand, and extrusion‐based deposition strategies.^[^ ^2^ ^]^ The printing of hydrogel precursors in extrusion‐based printing is an especially versatile and promising tool due to its compatibility with embedding baths, which are typically composed of a viscous liquid^[^ ^3^ ^]^ or a granular liquid‐like solid.^[^ ^4^ ^]^ Deposition of (bio)ink into these embedding/support baths allows for the free‐form fabrication of complex 3D structures. Using cross‐linkable embedding baths enables the biofabrication of multi‐scale and multi‐material constructs.^[^ ^5^, ^6^ ^]^ However, the current dependence on viscous liquids or granular liquid‐like solids imposes strict limitations on bioprinting versatility (e.g., low printing speeds, high bio‐ink viscosity, high cell death, and a limited amount of compatible materials), which hinders widespread clinical and industrial application.

Extruding bioinks with high viscosity associates with lowered cell viability due to high shear stresses within the nozzle, whereas the lower viscosity inks associate with poor printing fidelity.^[^ ^7^ ^]^ Shear‐thinning formulations have been investigated as a potential solution, but are still relatively viscous, compatible with only a restricted number of materials, and often reliant on added secondary materials to obtain shear‐thinning properties, or complex chemical functionalization of the material backbone (e.g., to introduce guest‐host interactions).^[^ ^8^, ^9^, ^10^, ^11^, ^12^ ^]^ Moreover, in current embedded printing approaches the strand size is dictated by the nozzle's inner diameter. These diameters are unfortunately relatively large as the shear stress inversely correlates with nozzle diameter owing to the ink's viscosity, which imposes strict limits on the printing speed versus printing resolution balance. Embedded bioprinting with low viscous bioinks is anticipated to break through these conventional limitations, however, no suitable strategy to achieve this has been reported to date.

Aqueous two‐phase systems (ATPS) can stabilize the interface of a large variety of aqueous polymer solutions in a versatile manner. Within ATPS, two structurally different polymers in an aqueous solution separate and form two stable and equilibrated phases.^[^ ^13^ ^]^ Despite its potential, ATPS has remained underexplored in biofabrication strategies with only a limited number of pioneering studies on introducing microporosity^[^ ^14^ ^]^ or on the fabrication of tube‐like structures via interfacial complexation.^[^ ^15^, ^16^ ^]^ However, ATPS has remained unstudied for the fabrication of living matter using embedded bioprinting.

In this study, we introduce ATPS stabilized low viscosity 3D (LoV3D) bioprinting for freeform fabrication of living tissue constructs. Within this study, we refer to all inks and baths that have a viscosity of < 100 mPas at relevant extrusion shear rates as being low viscous, which is in line with current nomenclature in literature and industry (Table S1, Supporting Information). Controlling ink and bath density enabled the 3D shape‐stable deposition of low viscous inks. This approach permitted independent bulk and ink crosslinking, and as no immediate ink crosslink is necessary LoV3D printing can be performed with a variety of crosslinking strategies. The liquid nature of ink and bath during printing further allowed for the printing of smooth interconnections of printed filaments. The diameter of printed strands was independently controlled by print speed and ink flow rate, allowing for on‐the‐fly tuning of print diameter offering print resolutions from the millimetric down to single‐cell resolution strands using a single continuously‐flowing nozzle. Unlike conventional print strategies that rely on more viscous inks, cell viability for all printing conditions was not compromised by shear stresses even at exceedingly high print rates due to the use of a low‐viscosity bioink. Moreover, it was demonstrated that cells and bioactive moieties could be attached (e.g., via discrete on‐cell crosslinking)^[^ ^17^ ^]^ directly onto the channel wall in a single‐step manner at significantly higher efficiencies than conventional solid/liquid systems owing to LoV3D's liquid/liquid nature. In short, we present an innovative ATPS stabilized all‐aqueous printing technique that is readily compatible with a wide variety of polymers, especially those that possess low viscosity, and demonstrated that LoV3D offers unique enabling advantages as compared to conventional printing approaches.

## 2 Results and Discussion

**Figure 1:**
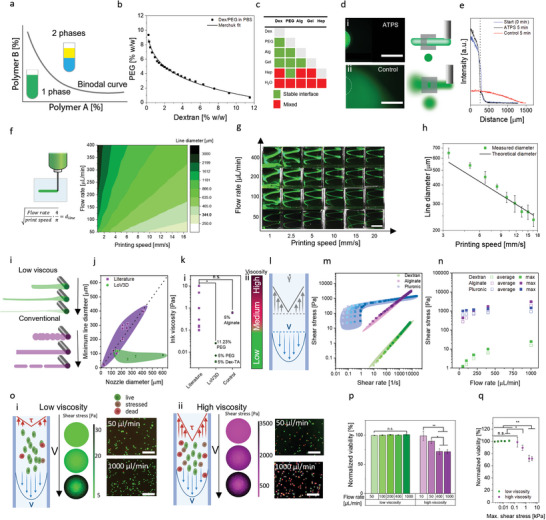
LoV3D enables cytocompatible high‐speed printing of low viscous polymer solutions over a wide range of resolutions. a) Schematic depiction of a binodal curve with indicated regimes. b) Binodal curve of a PEG/dextran system with Merchuk fit. c) Polymer combinations forming a stable (green) or unstable (red) interface. The stability was defined as a sharp interface after at least 60 min of incubation. d) 5% dextran + dextran‐FITC solution was printed in: i) 11.23% PEG bath and ii) PBS bath and fluorescently imaged after 5 min of incubation. The initial interface is indicated by the white dotted line. e) Intensity profile of the interface after 5 min for the ATPS (black) and control (red) system. f) Schematic depiction of ink deposition stating the influence of printing parameters onto the strand diameter with the range of theoretically achievable line diameters with a 25‐gauge nozzle at selected flow rates and print speeds. g) False‐colored spiral prints obtained with varying printing speeds and flow rates (5% PEG + 1 mg mL^−1^ RhodamineB in 1% w/w alginic acid). h) Correlation of theoretical and experimental diameters with 50 µL min^−1^ (*N* = 15). i) Schematic of strand thinning of low viscous (green) and viscous (purple) inks. j) Minimum diameters reported for several nozzle sizes in literature^[^ ^6^, ^24^, ^25^, ^26^, ^27^, ^28^, ^29^, ^30^, ^31^, ^32^, ^33^, ^34^, ^35^ ^]^ compared to diameters achieved with low viscous (5% w/w dextran) inks (*N* = 3). Depicted regions indicate the lower cut‐off of strand diameters that have been reported in the literature (purple) or were experimentally assessed using LoV3D inks (green). k) i) Correlation of viscosities reported in the literature ^[^ ^6^, ^16^, ^24^, ^26^, ^28^, ^30^, ^34^, ^35^ ^]^ with the inks employed in LoV3D printing and the utilized control (5% w/w alginic acid, purple) and ii) the responding nomenclature. l) Schematic depiction of velocity and shear rate profiles within a nozzle during extrusion. m) Shear stress of dextran (green), alginate (purple), and Pluronic‐127 (blue) at varying shear rates modeled at flow rates from 50–1000 µL min^−1^ (light to dark). n) Maximum (filled) and average (half‐filled) shear stresses during extrusion at various flow rates with dextran (green), alginate (purple), and Pluronic‐127 (blue). o) Schematic depiction of shear stresses within a nozzle together with shear stress profiles obtained from flow modeling and associated survival/death of 3T3 cells for i) low viscosity (5% w/w dextran) and ii) high viscosity (5% w/w alginate) ink extruded at various rates. p) Cell viability of 3T3 cells post‐extrusion, which was normalized to non‐extruded inks at several flow rates through a 25G nozzle (*N* = 3), and q) correlation of experimental cell viability with modeled maximal shear rates within the nozzle for the low viscous ink (5% w/w dextran, green) and the high viscosity control (5% w/w alginate, purple) (*N* = 3). Significance is indicated with ** *p* < 0.01, * *p* < 0.05, and n.s for *p* > 0.05, one‐way ANOVA with Tukey post hoc test. Scale bars: d) 1 mm, g) 10 mm, and o) 250 µm.

We postulated that embedded bioprinting of low viscous solutions would be enabled by ATPS stabilization of the liquid/liquid interface. To identify whether two aqueous polymer solutions can form ATPS, and determine at which polymer concentrations this is achieved, a phase diagram containing the binodal curve of these solutions can be determined (**Figure** **1**a). In this study, the binodal curve was established via cloud‐point titration^[^ ^18^ ^]^ for a model system containing dextran and polyethylene glycol (PEG) (Figure 1b). We demonstrated that ATPS stability was not compromised by the presence of crosslinking agents (Figure S1, Supporting Information).

While the ability to form a stable ATPS is crucial for LoV3D bioprinting, this does not constrain its applicability as most commonly utilized (bio)polymers in tissue engineering are able to form ATPS.^[^ ^19^, ^20^, ^21^ ^]^ To confirm the universal applicability of our approach, the formation of a stable interface was investigated for several commonly used (bio)polymer systems including dextran, PEG, alginic acid, gelatin, and heparin. Here, several combinations of polymers resulted in a stable interface (Figure 1c and Figure S2, Supporting Information) and thus were considered suitable for ATPS printing applications. To further visualize the interface and shape stability of printed aqueous solutions, the liquid/liquid interface of a printed ATPS was compared to a printed non‐ATPS control (Figure 1d,e). Dextran containing dextran‐FITC was extruded in a PEG (ATPS) or PBS (control) bath, which revealed that the interface formed between ATPS model solutions was stable, while the non‐ATPS control showed diffusive destabilization (Figure S3, Supporting Information).

The diameter of printed lines with low viscosity could be calculated based on the extrusion rate and printing speed (Figure 1f). This suggested that an exceptionally wide range of line diameters could be printed using a single nozzle diameter by simply varying the print speed and extrusion rate (Figure 1f and Video S1, Supporting Information), which was empirically confirmed by printing spiral‐like patterns of various thickness in an ATPS stabilized embedding bath (Figure 1g,h and Video S2, Supporting Information). Indeed, LoV3D was able to print a wide range of diameters with a single nozzle, which breaks through the historical limitation of extrusion strategies where the minimal print diameter is dictated by the nozzle's inner diameter (Figure 1i,j).^[^ ^10^ ^]^ LoV3D thus enables high‐resolution printing in a nozzle‐diameter independent manner via large‐dimension strand‐thinning by enabling the use of polymers that possess orders of magnitude lower viscosity than those currently used in bioprinting (Figure 1k and Table S1, Supporting Information).

The viscosity of bioinks not only influences the shape fidelity and resolution of extruded strands but also dictates the experienced shear forces to which cells in the bioink are exposed during the extrusion process. Shear stress is typically positively associated with viscosity, and is highest near the nozzle's wall (Figure 1l).^[^ ^22^ ^]^ To compare the shear stresses in currently used inks and LoV3D‐enabled low‐viscosity inks, a theoretical model for a 260 µm inner diameter nozzle (25 gauge, Figure S4, Supporting Information) was developed. An alginate solution reflecting the viscosity of conventional inks (control indicated in Figure 1k) and 25% Pluronic PF‐127 as a commonly applied shear‐thinning bioink,^[^ ^23^ ^]^ were chosen as controls and compared to an ink representative for LoV3D, namely 5% w/w dextran. Analyzing shear stress under various shear rates revealed that the shear stress was at least two orders of magnitude lower for the low‐viscosity dextran solution as compared to pluronic and alginate solutions (Figure 1m,n). This difference in shear stress has significant practical implications, as low‐viscosity inks could be printed at high speeds without adversely impacting cell survival, (Figure 1o and Figure S5, Supporting Information), which was in sharp contrast to the conventional high viscous inks as demonstrated with 3T3 mouse fibroblast (Figure 1p). Impressively, LoV3D printing was able to extrude at 1.8 m s^−1^ (e.g., 1000 µL min^−1^) through a nozzle with a 108 µm diameter hollow core (32 gauge) while maintaining > 96% cell survival (Figure S6, Supporting Information). When correlating cell viability with shear stress, it became evident that only polymer solutions with viscosities lower than those conventionally explored were suitable for high extrusion rates (Figure 1q). Thus, LoV3D's ability to use low‐viscosity bioinks uniquely offers the possibility for high extrusion speeds, even at reduced nozzle diameters, without compromising cell viability. Hence, when printing cells within low viscous solutions cell viability is merely influenced by the utilized materials and their corresponding crosslinking strategies. Additionally, using low viscous inks could overcome the need for the commonly used high polymer concentrations to create viscous inks, thus enabling the creation of less dense and more porous hydrogel networks.

**Figure 2:**
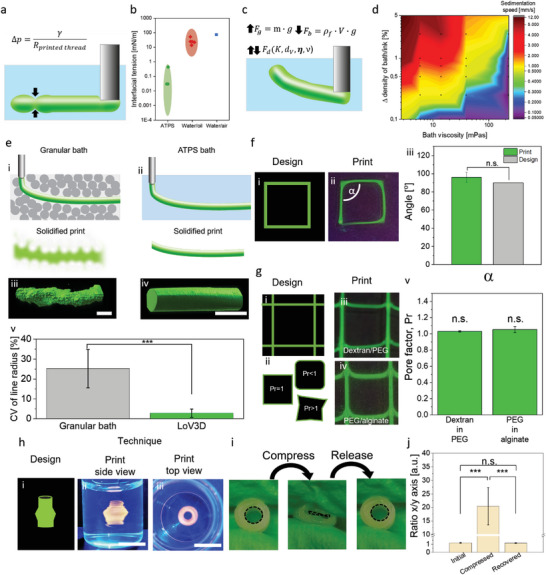
Physical parameters influencing ATPS print stability. a) Schematic depiction of droplet breakup induced by the Laplace pressure difference between bath and ink. b) Comparison of interfacial tension of liquid/liquid ATPS (green), water/oil (red) systems, and the water/air system.^[^ ^36^, ^37^, ^38^, ^39^ ^]^ c) Forces influencing the strand position and stability, which includes: gravitational force *F*_g_ influenced by the mass *m* of the solution as well as the gravitation acceleration *g*. Buoyancy *F*_b_ is influenced by the density of the matrix *ρ*_f_, volume of the ink *V*, and gravitation constant *g*. Drag force *F*_d_ is influenced by the drag coefficient *K*, the volumetric dimensions of the deposited ink *d*_v_, bath viscosity *𝜼*, and velocity *ν*. d) Sedimentation speed of a PEG droplet within a dextran bath with varying density mismatch and bath viscosity (*N* = 3). e) Comparison of low viscous ink behavior within i) granular embedding bath or ii) all‐aqueous LoV3D embedding bath. 3D reconstructions from confocal images of 5% w/w Dex‐TA (dextran‐tyramine) strands printed in iii) granular or iv) all‐aqueous LoV3D bath composed of 11.13% w/w PEG v) which were analyzed in the context of coefficient of variation (CV) of the strand radius, showing a significantly smoother strand when printed in all aqueous LoV3D surrounding (*N* ≥ 4). f) Single line fidelity assessment by comparing i) design with ii) print of fluorescently labeled dextran in PEG using fluorescent microscopy. iii) The angles (*α*) obtained showed no significant difference from the initial design (*N* = 4). g) Grid pore fidelity assessment by comparing i) design utilizing the ii) pore factor as a measure. iii,iv) Fluorescent micrographs of printed grids with dextran in PEG and PEG in alginate, respectively. v) Pore factor analysis of the obtained grids shows no significant differences between both ink/bath combinations and the design (*N* = 3). h) Printed 3D structure created with PEG as ink within an alginate bath based on i) 3D design. ii) Side view and iii) top view photographs of the created print within the bath. i) Photographs of a crosslinked and from the bath extracted 3D construct that was mechanically deformed using cyclical compression. j) Quantitative image analysis of the aspect ratio of channel cores before, during, and after compression (*N* = 3). Scale bars: e, iii, iv) 500 µm, h) ii,iii) 5 mm. Significance is indicated with *** *p* < 0.005 and n.s for *p* > 0.05, one‐way ANOVA with Tukey post hoc test.

To permit to use of low‐viscosity inks in embedded bioprinting, shape stability and spatial stability of strands extruded within the bath have to be ensured. The interfacial tension between the ink and bath phase can break up the printed strand into droplets, which limits conventional extrusion bioprinting approaches. The Young–Laplace equation specifies that the pressure difference resulting in the breakup depends on the interfacial tension as well as the diameter of printed strands (**Figure** **2**a). Assuming a cylindrical strand, with a length *R_1_* much larger than the radius *R_2_* the Young–Laplace equation (Equation (1)) can be adapted as follows:

(1) $$\Delta P=\gamma \left(\frac{1}{R_{1}}+\frac{1}{R_{2}}\right)\approx \gamma \frac{1}{R_{2}}$$

The radius of the strand *R_2_* and the interfacial tension between ink and matrix *γ* are parameters that destabilize the printed strands by increasing the pressure difference Δ*P*. Advantageously, when compared to water/air (72 mN m^−1^) or water/oil interfacial tensions (1–40 mN m^−1^),^[^ ^36^, ^37^ ^]^ the interfacial tension of ATPS is drastically lower (10^−4^–0.44 mN m^−1^)^[^ ^38^, ^39^ ^]^ (Figure 2b). These comparably low interfacial tensions, being a key feature of ATPS systems,^[^ ^13^ ^]^ result in a remarkably lower pressure difference that requires a substantially longer time interval before droplet breakup will occur. Importantly, this offers ATPS‐embedded bioprinting strategies with an ample window of time for deposition of the programmed bioink design prior to the occurrence of printed strand destabilization.

Another factor that can destabilize the design of printed strands is gravitational movement. Here, an intricate interplay between gravitational force, buoyancy, and viscous drag influences the strand position and sedimentation/rising speeds (Figure 2c). To explore the influence of these physical forces, the sedimentation speed of 20 µL droplets composed of PEG with varying concentration was mapped for a range of bath viscosities (dextran‐based bath with varying concentration) as well as ink/bath density mismatches (Figure 2d). As expected, sedimentation speed was positively correlated with density mismatch, and inversely correlated with bath viscosity. Furthermore, the sedimentation/rising speed correlated with the overall weight (volume) of the deposited strand, which is in line with the force equilibrium deviation. Generally, when printing with low viscous solutions in low viscous baths, the relative densities of ink and bath need to be equilibrated. This can be achieved by changing the ink or bath polymer concentration or polymer backbone, or via the addition of additives. Thus, utilizing low‐viscosity inks could require facile density tuning (Figure S7, Supporting Information), while obviating the need for the extensive rheological tuning that is commonly needed for conventional 3D printable (bio)inks.

To assess the fidelity of prints created within all aqueous solutions, we compared strands printed within conventional gelatin‐based granular baths with strands printed using LoV3D printing (Figure 2e). Prints extracted from the granular bath (Figure 2e‐iii) showed a rougher surface compared to prints from the LoV3D bath (Figure 2e‐iv). Comparing the coefficient of variation (CV) from the line radii showed a significant difference between granular and aqueous baths, providing a clear indication of the improved smoothness and monodispersity of strands printed with LoV3D (Figure 2e‐v and Figure S8, Supporting Information). The compliance of original designs versus printed single lines was assessed (Figure 2f (i,ii)), which confirmed high print fidelity as no significant differences were measured (Figure 2f,‐iii). Furthermore, the print fidelity of 2D structures (grids) was also assessed using the pore factor *Pr* ^[^ ^40^ ^]^ (Figure 2g (i,ii)). Two ink and bath combinations (e.g., dextran in PEG and PEG in alginate) were investigated (Figure 2g (iii,iv)), and for both the *Pr* was close to one, and no significant differences in *Pr* were measured as compared to the original design (Figure 2g‐v). Together this indicated the high printing fidelity nature of LoV3D printing. Similar to embedded bioprinting techniques that rely on highly viscous or liquid‐like solid baths, LoV3D was readily compatible with the fabrication of 3D constructs (Figure 2h), which closely resembled the design and further could be removed from the bath subsequent to crosslinking (Figure S9a, Supporting Information). Printed structures were compressed and upon force, release recovered immediately to 98 % ± 3.5 % of their initial shape (Figure 2i). Impressively, structures showed no significant difference in channel aspect ratio before and after compression (Figure 2j), thus providing high shape fidelity even after severe and repeated mechanical deformation. Moreover, following compressive deformation, the printed construct did not reveal any sign of layer delamination, which was corroborated by the absence of fluid leakage when being perfused (Figure S9b, Supporting Information).

**Figure 3:**
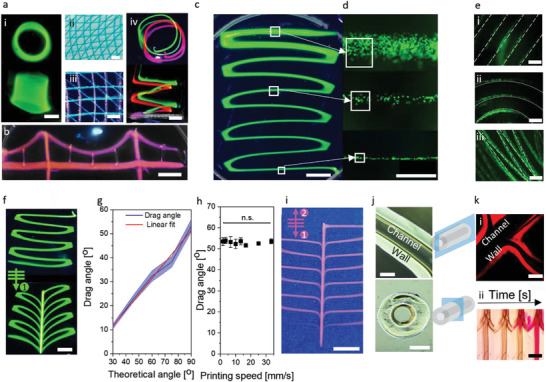
LoV3D allows for a manifold of printing shapes while filament thinning enables high‐resolution printing, and aqueous‐filament merging enables the printing of smoothly interconnected lines and channels. a) Fluorescent microphotographs of printed i) tube, ii,iii) grid, and iv) spiral‐shaped structures to demonstrate ATPS print stability at various shapes and the ability for multi‐material printing. b) Photograph of a bridge design was printed in a continuous manner to demonstrate LoV3D's ability to print interconnected lines of various line diameters using a single nozzle. c) Fluorescent microphotographs showing the thinning ability of liquid printed strands enabling d) the pattering of cells (e.g., green‐labeled 3T3 cells) down to single‐cell resolution. e) Fluorescent microphotographs of Draq5 labeled 3T3 cells (green) in a Dex‐TA ink printed within a gelatin matrix demonstrating the feasibility of the printing of adjacent cell‐laden lines offering the ability for cell patterning. f) Fluorescent microphotographs of a linear serpentine line that was connected via a central axis via a monodirectional drive‐through motion (indicated in schematic, top left) of the nozzle to create a complex curved interconnected structure. g) Dependency of the drag angle (*N* = 3) to the initial (theoretical) angle was calculated and empirically measured using fluorescent microscopy and fitted with an R^2^ of 0.987. h) Drag angle at different printing speeds of the central line showed no significant difference (*N* = 3). i) Fluorescent microphotographs of a linear serpentine line that was connected via a central axis via a bidirectional drive‐through motion (indicated in schematic, top left) of the nozzle to create a complex interconnected structure without major curve deformation. j) Brightfield microphotographs of a tube created by a hydrogen peroxide containing sacrificial PEG ink within a horseradish peroxide containing Dex‐TA bath via inside‐out crosslinking with schematic depictions of imaging planes. k) Creation of branched tubes with i) confocal fluorescent microphotograph of a branched tube showing channel interconnectivity allowing for ii) perfusion of the created canicular structures. Significance is indicated with n.s for *p* > 0.05, one‐way ANOVA with Tukey post hoc test. Scale bars: a) iiv), c), f) 5 mm, a) ii), k) ii) 2 mm, a) i) 1 mm, b) 10 mm, d), e), i), j), k) i) 500 µm.

Next, we investigated LoV3D's ability to print complex 3D structures. Here, the alginate/PEG model system using 5% w/w PEG as ink and 1% w/w alginate as the bath was employed. The suitability of LoV3D printing to create 3D shapes was demonstrated for common complex structures such as tubes and grids (**Figure** **3**a (i,ii) and Video S3, Supporting Information). Furthermore, the ability to create multi‐material prints was demonstrated using grid‐like and intertwined spiral structures (Figure 3a (iii,iv)). The thinning ability of low viscous ink enabled prints with the facile interconnection of aqueous strands with various diameters utilizing only a single nozzle (Figure 3b). The print diameter could be inline controlled by adjusting the printing speed for both interrupted and continuous lines (Figure 3c). Interestingly, this thinning behavior could be leveraged for cell placement by uniquely enabling ultra‐high resolution prints creating strands containing aligned cell trains at single cell width, which thus allowed for deterministic cell placement in the *x*‐*z*‐plane using conventional large diameter nozzles (Figure 3d and Figure S10, Supporting Information).

Being able to spatially arrange distinct cell types is an important feature of 3D bioprinting, as it allows for the replication of more complex and native‐like tissue structures. LoV3D also allows for high‐fidelity printing of adjacent lines within a cell‐laden bath. To demonstrate this, parallel lines, crossing zig‐zag lines, and concentric rings of increasing curvature were printed, which could be printed without adversely affecting neighboring lines despite the low viscous nature of the materials (Figure 3e). This feature in combination with earlier demonstrated multi‐material printing could offer a facile method for multi‐cell type or multi‐material patterning.

As LoV3D printing is suitable for a variety of crosslinking types, the printing of sacrificial inks was explored. Here, the low viscosity of the inks offers the advantage of facile ink removal after printing without the need for, for example, temperature changes to achieve a solid/liquid transition.^[^ ^6^, ^35^ ^]^ Sacrificial inks are most commonly used to create perfusable networks, however, creating smooth, interconnected, and complex, channel networks has remained challenging as liquid‐like solids do not readily allow for the physical integration of printed lines. With LoV3D printing several parallel printed lines could be effortlessly fused into complex shapes via a “drive‐through” motion of the nozzle (Figure 3f and Video S4, Supporting Information), which for example allows for the creation of smooth monolithic structures with a central strand and multiple side branches. The angle at which the side branches are connected (e.g., drag angle) was directly controlled by the angle at which the newly deposited strand was driven through the initial strand (Figure 3g and Figure S11, Supporting Information). While the printing speed determined the channel diameter, it did not influence the drag angle of the printed structures, which thus allows for independent tuning of the curvature and diameter of the printed complex channel structures (Figure 3h). Consequently, the drag of the printed line can also be fully reversed by simply applying the same deformation in a reciprocal manner via a double drive‐through (back and forth) motion, which offers a feat potentially unique to low viscous inks (Figure 3i).

To investigate the suitability of LoV3D's smoothly fused interconnections for the creation of perfusable channel networks, a sacrificial PEG ink containing a cytocompatible level of H_2_O_2_ was printed into a Dex‐TA bath containing the enzyme horseradish peroxidase (HRP). The diffusion of H_2_O_2_ from the ink into the bath initiated enzymatic covalent crosslinking of tyramine moieties, which resulted in the formation of a conformal hydrogel shell around the ink effectively creating tubular structures with a centered circular aqueous channel (Figure 3j). Here, the main channel could be connected with complex‐shaped side channels (Figure 3k‐i) that were directly perfusable (Figure 3k‐ii and Video S5, Supporting Information). hMSCs incorporated within the Dex‐TA ink showed the viability of 93.1 % ± 3.4 % at day 7 (Figure S12, Supporting Information) and, hence, proved LoV3D printing as being cytocompatible. Overall, LoV3D bioprinting allows for the creation of a variety of prints ranging from 2D stacked lattices to 3D spirals to interconnected complex hollow structures. Moreover, the thinning ability of the low viscous ink enabled high‐resolution prints even when using large nozzles making LoV3D a low‐shear, high‐resolution, and high speed printing technique. While prints created by covalent stabilization of the ink are part of the possible application fields, LoV3D printing is anticipated to be particularly suitable for sacrificial printing approaches. Here, the bulk (crosslinked bath material) forms the final structure with, for example, patterned channels, cells, or other materials within. Enabling the use of unmodified bulk precursors allows for a large variety of bulk materials that can potentially be employed. Overall, this enables the creation of relatively large constructs with embedded patterns within a short time frame, as merely the pattern has to be printed. Hence, the stability of the system has to be warranted for a comparably short time frame.

**Figure 4:**
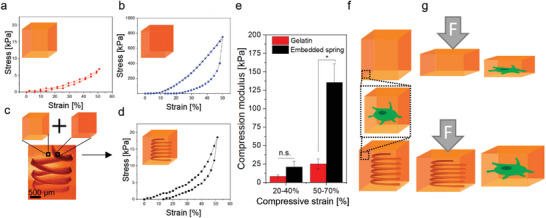
Printing mechanical hydrogel springs in hydrogel constructs using LoV3D allows for uncoupling the mechanical properties of the cellular level and the tissue level. Stress‐strain curves obtained from compression tests of a) gelatin bulk, b) PEGDMA and alginate bulk, or c,d) gelatin bulk containing a spring of PEGDMA and alginate. e) Compression moduli calculated in a low strain (20–40%) regime and high strain (50–70%) regime of the pure gelatin bulk (red) as well as with a PEGDMA alginate spiral printed within the bulk. f) Illustration indicating that incorporated cells within the gelatin, as well as the composite, would on the micro level only be surrounded by gelatin. g) Schematic illustration that traumatic external forces can potentially be counteracted by the printed internal hydrogel spring, which mechanically protects both cells and constructs. Significance is indicated with * *p* < 0.05 and n.s. *p* > 0.05, 1‐way ANOVA with Tukey post hoc test.

In addition to sacrificial printing either within a sacrificial bath or printing of a sacrificial ink, we envision LoV3D printing to be a facile tool to combine materials with a broad range of mechanical properties post‐printing. While common extrusion printing relies on the intricate interplay of ink and bath properties as well as rheological tuning, for example, by additive addition, limiting the number of material combinations, LoV3D is postulated to overcome this constraint. As LoV3D printing does not require rheological adaption and can be applied to a large variety of commonly applied (bio)polymers, we envision the facile combination and patterning of materials with desired mechanical properties post‐crosslinking. To investigate this hypothesis, we aimed to combine two ATPS‐forming polymers with orders of magnitude difference in their compression moduli, which both could be stabilized via photocrosslinking. Gelatin was chosen as a photocrosslinkable, cytocompatible, and post‐crosslinkable mechanically soft bath with a compression modulus of 8.1 ± 2.4 kPa in the low strain regime (20–40% strain) (**Figure** **4**a). As a stiff material, poly(ethylene glycol)‐di‐methacrylate (PEGDMA) and alginate were combined as a dual crosslinkable hydrogel. The resulting material was photocrosslinkable utilizing the methacrylates present at the PEGDMA backbone. Ionic post‐stiffening via physical crosslinking of alginate chains in the presence of Ca^2+^ resulted in a compression modulus of 1796.1 ± 174.8 kPa in the low‐strain regime (Figure 4b). The materials were combined by printing a PEGDMA and alginate spring within a gelatin bath. Subsequently, both materials were photocrosslinked and the alginate was stiffened by incubating the resulting construct in a CaCl_2_ solution. Interestingly, by combining these two material systems (Figure 4c,d), the resulting construct contained an internal spring that enabled stiffening of the bulk upon extensive deformation (> 40% deformation). Specifically, a slightly increased compression modulus of 21.2 ± 7.2 kPa was observed for the composite in the low strain regime (Figure 4e), while at high strains the impact of the embedded spring became prominent, resulting in a five‐fold higher compression modulus of the composite (135.4 ± 25.4 kPa) as compared to pure gelatin (25.2 ± 6.8 kPa). We hypothesize, that such a multi‐material design could potentially allow cells that are incorporated within the bulk to exclusively sense a soft microenvironment (Figure 4f), while the embedded spring offers a potential solution for damping traumatic external forces at the construct level (Figure 4g). Overall, due to its applicability to low viscous solutions, LoV3D bioprinting allows for the use of rheologically unmodified precursor solutions. Therefore research efforts can focus on the final mechanical properties of the crosslinked construct. Consequently, LoV3D multi‐material bioprinting allows for constructs with designer mechanical behavior with emergent properties by combing very soft and stiff materials within a single construct.

**Figure 5:**
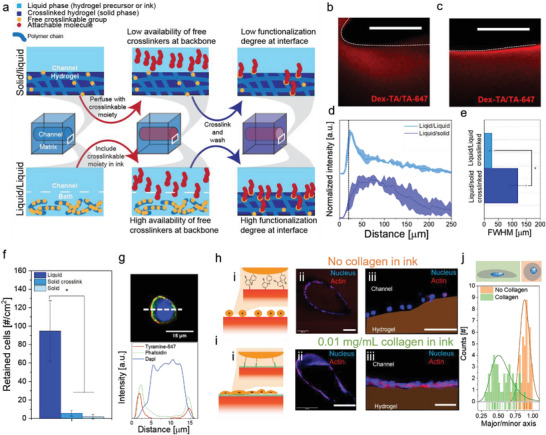
Liquid/liquid interfaces enable more efficient surface modifications as compared to traditional solid/liquid interfaces. a) Schematic depicting the functionalization of solid/liquid (top) and liquid/liquid (bottom) interfaces indicating higher functionalization efficiency at liquid/liquid interfaces, which is attributed to higher availability of reactive groups. Confocal fluorescent microphotograph of a printed channel cross‐section after perfusion and TA‐binding dye attachment at a b) solid/liquid interface and c) liquid/liquid interface, dotted lines indicate channel wall position. d) Intensity profile plots of solid/liquid and liquid/liquid interfaces starting at the channel wall (black dotted line) into the hydrogel bulk, and the e) full width half maximum (FWHM) of the measured peaks (*N* = 3). f) Amount of retained cells that were seeded (in presence of crosslinkers) onto a liquid/liquid or solid/liquid interface. g) Confocal fluorescence microphotograph (top) of a cell with attached TA‐647 (red) stained with DAPI (blue) and phalloidin (green) after fixation with respective cross‐section intensity plots (bottom). Channels printed in Alginate‐TA with 4 × 10^7^ cells mL^−1^ h) without collagen or i) with 0.01 mg mL^−1^ collagen in the ink. i) Schematic of cell attachment onto the printed hydrogel and confocal fluorescence microphotographs of ii) cross‐sectional, iii) magnified confocal images of printed channels stained with Dapi (blue) and phalloidin (red) after five days of culture. j) Nucleus shape analysis of confocal images obtained without collagen in the ink (orange) or 0.01 mg mL^−1^ collagen (green). The ratio of the major and minor axis indicates the sphericity of the nucleus. Significance is indicated with * *p* < 0.05, one‐way ANOVA with Tukey post hoc test. Scale bars: b), c), h) iii), i) iii) 50 µm, h) ii), i) ii) 250 µm.

In contrast to liquid‐like solid printing systems, LoV3D has a fully liquid nature, which we postulated would be advantageous to facilitate reactions and bonding at the liquid/liquid interface as compared to liquid‐solid interfaces. Specifically, in traditional solid‐liquid systems, many of the reactive groups have become bound or inaccessible due to the crosslinking process. In contrast, all reactive groups are still available at LoV3D's liquid/liquid interface, which should therefore present the reactive groups at higher concentrations for functionalization (e.g., incorporation of cell‐instructive moieties) (**Figure** **5**a). To confirm this hypothesis, sacrificial PEG ink was printed in an alginate‐TA bath. For the solid/liquid interface, the bulk was crosslinked post‐printing and subsequently perfused with a dye containing a fluorescent crosslinkable moiety (TA‐647) together with photo‐crosslinkers. Successful tyramine‐tyramine bonding of the dye was confirmed by confocal imaging (Figure 5b,c, and Figure S13, Supporting Information). For LoV3D's liquid/liquid system, the dye was directly incorporated into the ink during the printing, which is associated with a much higher intensity of bound dye as compared to the solid/liquid system. This indicated that LoV3D's liquid/liquid nature indeed allows for more efficient functionalization than conventional solid/liquid print approaches (Figure 5d). Notably, liquid/liquid interfaces resulted in a more localized crosslinking at the interface when compared to the solid/liquid system. This was corroborated by the full width half maximum (FWHM) of the intensity curves, which indicates a more controlled and localized functionalization (Figure 5e). LoV3D, therefore, represents a novel method to increase the efficiency of channel functionalization, while introducing a novel one‐step solution for channel printing and coating. This strategy could potentially be adapted to other surface functionalization fields such as hydrogel‐based sensing.^[^ ^41^ ^]^

Recently, Kamperman et al.^[^ ^17^ ^]^ introduced discrete inducible on‐cell crosslinking to enable local and temporally‐controlled tethering of cells onto hydrogels via an enzymatic tyramine‐tyrosine crosslinking. We explored this reaction to test our hypothesis that liquid/liquid interfaces are more efficient reactive substrates than conventional solid/liquid interfaces. To this end, a PEG solution containing 1 × 10^7^ 3T3 fibroblasts mL^−1^ was deposited on top of a liquid alginate‐TA (liquid/liquid interface) or crosslinked alginate‐TA bath (liquid/solid interface) in the presence of an activated photocrosslinker. Following vigorous washing of the hydrogel surfaces, significantly more cells were still adhered to the hydrogel when crosslinked at liquid/liquid interfaces as compared to solid/liquid interfaces (Figure 5f and Figure S14, Supporting Information). This further substantiates the claim that liquid/liquid interfaces can be exploited to enhance the efficiency of interface functionalization. To confirm that cells were indeed able to form covalent bonds with the tyramines of the polymer, TA‐647 dye was demonstrated to be able to crosslink onto the surface of cells (Figure 5g). Subsequently, cells were incorporated into a sacrificial PEG ink to demonstrate that tyramine‐tyrosine bonding could be leveraged to enable a one‐step procedure to adhere cells onto the channel walls during the printing of a 3D construct in a cytocompatible manner (Figure 5h and Figure S15, Supporting Information). The cells were revealed to be bound evenly over the channel wall but did not spread over time as they remained spherical over a culture period of five days, which corroborates that after cessation of crosslinking, tethered cells were unable to readily make novel bonds to the material's surface. Indeed, when collagen was incorporated in the sacrificial ink, which allows the collagen's tyrosines to covalently bond with the polymer's tyramines, tethered cells were able to not only attach, but also spread out over the hydrogel's surface (Figure 5i). Comparing the nuclear shape of cells printed in the presence or absence of collagen revealed that the addition of cell adhesive domains (e.g, collagen's RGDs) significantly elongated the nucleus of bound cells (Figure 5j). This demonstrated that LoV3D can be used to either discretely tether cells to otherwise non‐adhesive biomaterials with unprecedented efficiency, while simultaneously allowing for efficient single‐step chemical modification of printed channels to guide both cell shape and behavior (e.g., cell attachment and spreading). This approach can offer an elegant and versatile solution for the creation of (complex) tissues including those containing intricate vascular channel networks.

## 3 Conclusion

We here introduce LoV3D bioprinting as a highly versatile and rapid bioprinting technique that opens a new window of 3D bioprinting capabilities. This technique is inherently compatible with an unprecedentedly wide range of biomaterials that are commonly utilized in tissue engineering applications. Specifically, LoV3D enables the use of inks that are of substantially lower viscosity than currently employed inks, which enabled high‐resolution prints without compromising printing speed or cell viability due to reduced shear stress. Notably, the use of low‐viscosity biomaterials uniquely allowed for the large dimensional thinning of printed strands allowing for high‐resolution prints of (cell‐laden) fibers down to single‐cell resolution. Exploiting distinct crosslinking techniques, LoV3D enables the creation of designer filaments, patterned bulk multi‐materials, tubes, and complex‐shaped perfusable structures. Importantly, LoV3D's use of liquid/liquid interfaces offers a versatile platform for single‐step surface functionalization and cell seeding at substantially higher efficiencies than conventional multi‐step print‐and‐coat/seed approaches. In short, we demonstrated that LoV3D printing is a versatile tool that offers numerous unique benefits for the biofabrication of hydrogel constructs and living matter.

## 4 Experimental Section

### Materials

Dimethylformamide (DMF, 99.8% anhydrous), 4‐nitrophenyl chloroformate (PNC, 96%), tyramine (TA, 98%), polyethylene glycol (35 000), heparin sodium salt (from porcine intestine mucosa), dimethyl sulfoxide‐d6 (DMSO‐d_6_), fluorescein sodium salt, RhodamineB, dextran‐FITC (40 kDa), horseradish peroxidase (HRP, 250 U mg^−1^), hydrogen peroxide (H_2_O_2_, 30% w/w), calcein AM, ethidium homodimer‐1 (EthD‐1), L‐Proline, ascorbic acid 2‐phosphate (ASAP), Triton X‐100, fetal bovine serum (FBS), gelatin (gel strength 300, type A) were purchased from Sigma Aldrich/Merck. Tyramine hydrochloride (99%) was purchased from Acros Organics. Dextran (500 kDa), pyridine (99.8%), and lithium Chloride were purchased from Alfa Aesar. Tyramide‐AlexaFluor647 (AF647) and 4′,6‐diamidino‐2‐phenylindole (DAPI), 2″‐[4‐ethoxyphenyl]‐5‐[4‐methyl‐1‐piperazinyl]‐2,5″‐bi‐1H‐benzimidazole) (NucBlue, Hoechst), 1,5‐bis{[2‐(di‐methylamino) ethyl]amino}‐4, 8‐dihydroxyanthracene‐9,10‐dione (DRAQ5), non‐essential amino acids (NEAA), and Phalloidin‐AF647 were purchased from Fisher Scientific. Dulbecco's modified Eagle's medium (DMEM), alpha modification of Eagle's medium (*α*MEM), penicillin and streptomycin, 2‐mercaptoethanol, GlutaMAX, and trypsin‐EDTA were purchased from Gibco. Rat tail Collagen type I was purchased from Corning. Dextran (40 kDa, EP) was purchased from Pharmacosmos. Alginate (80–120 cps) was purchased from FUJIFILM Wako Chemicals Europe. 4‐(4,6‐Dimethoxy‐1,3,5‐triazin‐2‐yl)‐4‐methylmorpholinium chloride (DMTMM) was purchased from Fluorochem. The ruthenium/sodium persulfate (Ru/SPS) crosslinking kit was purchased from Advanced BioMatrix. Polyethylene glycol dimethacrylate (PEGDMA, *n* = 14) was purchased from TCI chemicals. Calcium chloride dihydrate and ethanol were purchased from Supelco. Phosphate‐buffered saline (PBS) was purchased from Lonza. Hydrolyzed gelatin type A (125.8 kDa, Rousselot #: 12214078APS10A1) was kindly provided by Rousselot Biomedical.

### Material Synthesis

Dextran‐tyramine (Dex‐TA) was synthesized as previously described.^[^ ^42^ ^]^ The ^1^H‐NMR analysis confirmed successful functionalization with the final polymer containing 4.4 tyramine moieties per 100 repetitive monosaccharide units (Figure S16, Supporting Information). Alginate‐tyramine (Alginate‐TA) was synthesized via a one‐step route. Here, alginic acid sodium salt (80–120 cps) (1 g) was dissolved in MilliQ water (500 mL). Subsequent to dissolution, DMTMM (6.9 g, 50 mm) and tyramine HCl were added (4.34 g, 50 mM), and allowed to react for 48 h. The solution was then precipitated in ice‐cold ethanol, filtered, and vacuum dried. The product was dialyzed against MilliQ water (Spectra/Por, MWCO 1 kDa) for five days and subsequently lyophilized. The product was analyzed via UV–vis spectroscopy (NanoDrop ND‐1000 spectrophotometer, 275 nm) and contained 4.9 tyramine moieties per 100 repetitive units.

### Cloud‐Point Titration

The binodal curve of poly(ethylene glycol) (PEG, 35 kDa) and dextran (40 kDa) was established via cloud‐point titration following the procedure described by Kaul et al.^[^ ^18^ ^]^ Briefly, 15% w/w PEG in PBS was mixed dropwise into a 15% w/w dextran solution in PBS. When the solution turned opaque, the cloud point was considered to be reached. The added volume of the PEG solution was monitored by gravitational analysis. A measured volume of PBS was added until the system was returned below the binodal line and the solution regained transparency. This procedure was repeated until the binodal curve was established. Binodal curves were created with *N* = 3 titrations and confirmed with a Merchuk fitting (Equation (2)) following ^[^ ^43^ ^]^

(2) $$Y=M_{1}e^{\left(M_{2}X^{0.5}+M_{3}X^{3}\right)}$$

where *X* and *Y are* the polymer concentrations and *M_1_*, *M_2_*, and *M_3_* are fitting coefficients of the binodal curve.

### ATPS Stability

To determine interface stability between a variety of commonly used polymers, 10% w/w polymer solutions of PEG (35 kDa), 10% w/w of dextran (40 kDa), 10% w/w of heparin, 10% w/w of gelatin (hydrolyzed), or 2% w/w of alginic acid (80‐120 cps) were prepared in MilliQ water. The solutions were brought into contact and a clear interface after 60 min was considered stable (Figure S2, Supporting Information).

### Interface Stability ATPS versus Non‐ATPS

The interface of a 1 uL droplet of 5% w/w dextran and 1 mg mL^−1^ Dex‐FITC 40 kDa in 11.23% w/w PEG was observed over time and compared to a non‐ATPS control, where the PEG bath was replaced by PBS. The interface was monitored via fluorescent imaging. The intensity profile of the Dex‐FITC was obtained with Image‐J software (Figure S3, Supporting Information).

### Sedimentation Screening

To determine the sedimentation rate of droplets within a bath with varying viscosity, 20 µL droplets of varying concentrations of PEG solution to achieve 0%, 0.25%, 0.5%, 0.75%, 1%, 2.5%, or 5% density deviation from the dextran based bath solution were deposited in the respective bath. Sedimentation was calculated based on time‐resolved brightfield microscopy images (*N* = 3). Bath viscosity was tuned based on the concentration and molecular weight of dextran (40 and 500 kDa). The viscosities and densities of the utilized baths are listed in Table S2, Supporting Information.

### Rheology

Rheological analysis was performed using an Anton Paar‐Physica MCR 301 Rheometer (Anton Paar) using a parallel plate (PP) or cup (DC) setup. Stress‐strain curves were obtained with a strain of 1 mm min^−1^ in PP configuration. Viscosity measurements were performed using the DC configuration, screening the viscosity over a range of shear rates (0.01–1000 mm s^−1^). Measurements were performed with *N* = 3 samples.

### ATPS Printing

To demonstrate the feasibility and universal applicability of LoV3D printing, several polymer combinations were utilized. If not stated otherwise, all solutions were prepared in PBS and printed through a 25 gauge nozzle at a flow rate of 50 µL min^−1^. Printing was performed by combining an Inkredible+ printer (CELLINK) with a syringe pump (Harvard Apparatus, PHD Ultra). To assess the influence of flow rate and printing speed on the line diameter 5% w/w PEG (containing 1 mg mL^−1^ fluorescein sodium salt or RhodamineB for visualization) was printed within a 1% w/w alginate bath. Here, flow rates of 50, 100, 200, and 400 µL min^−1^ were combined with printing speeds of 1, 2.5, 5, 10, 15, and 20 mm s^−1^. Obtained prints were imaged under UV light and the line diameter was analyzed using ImageJ software (N = 3).

### Shape Fidelity Assessment

The shape fidelity of printed strands was analyzed on different length scales. To assess the line fidelity, we printed 5% w/w Dex‐TA containing 1 U mL^−1^ HRP and 1 mg mL^−1^ Dextran‐FITC (40 kDa) into an 11.23% w/w PEG bath containing 0.05% H_2_O_2_. For comparison with conventional strategies, a granular bath was created with gelatin granules (detailed description in supplementary information). Printed strands were collected from the bath and microphotographed using confocal microscopy. 3D reconstructed images were created using Imaris and the line radius distribution was assessed using ImageJ. The fidelity of single lines, as well as grids, was assessed by printing 5% w/w Dex‐TA into an 11.23% w/w PEG bath. Angle as well as the perimeter was assessed using ImageJ. The pore factor was calculated as follows from the perimeter *L* and the area *A* (Equation (3)): ^[^ ^40^ ^]^

(3) $$Pr=\frac{L^{2}}{16\cdot A}$$

### Crosslinking

Photocrosslinking was performed using a ruthenium (Ru) and sodium persulfate (SPS) based system. To this end, 1 mm Ru and 10 mm SPS or 1 mm Ru and 5 mm SPS in the presence of cells were used, and samples were irradiated for 120 s with visible light. Enzymatic crosslinking of Dex‐TA was performed in the presence of 1 U mL^−1^ HRP and 0.05% v/v H_2_O_2_.

For the printing of tubular structures 11.23% w/w PEG containing 0.05% H_2_O_2_ was printed within a bath composed of 5% w/w Dex‐TA containing 1 U mL^−1^ HRP. Diffusion of the crosslinker from the ink into the bath resulted in the formation of a hydrogel shell. After crosslinking, the print was removed from the bath and stored in PBS.

For the creation of a soft embedding bath, a 7.5 w/w% gelatin solution was utilized, exploiting the presence of photocrosslinkable tyrosines for stabilization. 20% PEGDMA mixed with 2% alginate was used as a dually crosslinkable ink. After printing, bath and ink were immediately stabilized via photocrosslinking and subsequently ionically crosslinked in a 1 m CaCl_2_ solution overnight.

### Cell Culture

3T3 mouse fibroblasts were cultured in a culture medium composed of DMEM containing 10% v/v FBS, 1% v/v, 100 U/ml Penicillin, 100 µg mL^−1^ Streptomycin (denoted as a full medium), and 1.4 µg mL^−1^ 1‐mercapto‐ethanol. Human mesenchymal stem cells (hMSCs) were cultured in a medium composed of *α*MEM containing 10% v/v FBS, 1% v/v, 100 U ml^−1^ Penicillin, 100 µg mL^−1^ Streptomycin, 1% v/v 20 mM ASAP, and 1% 100X GlutaMAX (denoted as a full medium). Human primary chondrocytes (hPCs) were cultured in a medium composed of DMEM containing 10% v/v FBS, 1% v/v of a combination of 100 U ml^−1^ Penicillin and 100 µg mL^−1^ Streptomycin, 1% v/v 20 mm ASAP, 1% v/v 35 mm L‐Proline, 1% v/v 100X NEAA, (denoted as a full medium). Cells were cultured at 37 °C under 5% CO_2_. Cells were detached when 80% confluency was reached using 0.25% Trypsin‐EDTA at 37 °C and kept at the required concentrations in DMEM on ice for ink preparation. Cell viability was assessed via incubation with 1.5 µm calcein AM (live) and 6 µm EthD‐1 (dead) in DMEM and imaged using fluorescent microscopy. For all cell‐containing prints, polymer solutions were prepared with full medium and samples were incubated in full medium subsequent to printing, if not stated otherwise.

### Cell Printing

To assess the viability of cells after extrusion at several flow rates, 3 × 10^6^ cells mL^−1^ of 3T3 cells were dispersed in DMEM containing 5 w/w% dextran, 5 w/w% alginate, or no polymer. Solutions were kept on ice and extruded through a 25‐gauge or a 32‐gauge nozzle (*N* = 3). The extrudate was collected and kept on ice. Subsequently, the samples were stained in a 48 wells plate with 1.5 µm calcein AM (live) and 6 µm EthD‐1 (dead) and imaged using an EVOS microscope. For comparison the viability was normalized to the viability within the syringe after the experiment to only assess shear‐related cell death and exclude all other external factors. Cell patterning was demonstrated by printing cells 3T3 cells stained with Draq5 (10 µm) into a bath containing cells stained with Hoechst (following supplier protocol). After staining, 3 × 10^6^ Draq5 labeled cells mL^−1^ were printed within a 5% w/w solution Dex‐TA prepared ink into a 7.5% w/w gelatin bath containing 3 × 10^6^ Hoechst labeled cells mL^−1^. Ink and bath were crosslinked via enzymatic crosslinking (Dex‐TA) and photocrosslinking (gelatin). The samples were imaged using an EVOS fluorescent microscope. Cell‐laden Dex‐TA lines were printed utilizing a Dex‐TA/PEG ATPS. Final inks had a concentration of 5% w/w Dex‐TA, 1 U mL^−1^ HRP, 10^6^ cells mL^−1^ (3T3, hMSC, or hPC) and were printed within an 11.23% w/w PEG bath containing 0.05% H_2_O_2_. Printed cell‐laden lines were extracted and cultured in full medium for up to seven days. On days 1, 3, and 7 the samples were stained with 1.5 µm calcein AM (live) and 6 µm EthD‐1 (dead) and imaged using an EVOS microscope (*N* = 10). Cell‐laden channels were printed utilizing an Alginate‐TA/PEG system. Here, 1% w/w Alginate‐TA served as a bath and 5% w/w PEG containing 4 × 10^7^ cells mL^−1^ 3T3 with or without 0.01 mg mL^−1^ collagen type I was used as ink. Lines were printed with 50 µL min^−1^ flow and the bath was photocrosslinked post‐printing. After crosslinking, the construct was easily handleable and compatible with conventional cell culture techniques. Here, prints were cultured in a full medium at 37 °C under 5% CO_2_ for up to five days. Subsequently, prints were fixated via incubation with 10% formalin at 4 °C for 30 min and subsequently kept in PBS until further use. For visualization, cells within prints were permeabilized using 0.2% v/v Triton‐X for 15 min and subsequently incubated with 2.5 U mL^‐1^ of Phalloidin‐AF647 for 2 h, and 1 mg L^−1^ of DAPI for 30 min.

### Cell Retention

Crosslinking‐induced cell attachment in liquid/liquid or solid/liquid interface systems was assessed by plating a 3% PEG solution containing 5 × 10^5^ cells mL^−1^ onto 1% alginate‐TA before or after photocrosslinking with a final cell density of 5 × 10^5^ cells per plate. To allow for crosslinking of seeded cells onto already pre‐crosslinked Alginate‐TA, 1 mm Ru, and 5 mm was added to the ink. All samples were irradiated using visible light for 90 s. Subsequently, all samples were imaged using brightfield microscopy, washed vigorously with PBS, and imaged again. Cell retention was analyzed by comparing the total amount of cells cm^‐2^ based on the obtained brightfield images (*N* = 3).

### Tyramide‐AlexaFluor647 (TA‐647) Attachment

The ability of cells to adhere to tyramine‐conjugated polymer backbones was assessed by reacting 3T3 mouse fibroblasts with TA‐647 enzymatically (3 U mL^−1^ HRP, 0.02 % w/w H_2_O_2_) or via photocrosslinking (1 mm Ru and 5 mm SPS, 90 s light irradiation). To cease the reaction, samples were diluted with a medium. Subsequently, cells were fixed via incubation in 10% formalin for 10 min. The fixed cells were stained via incubation with 2.5 U mL^−1^ of Phalloidin‐AF488 (2 h) and 1 mg L^−1^ of DAPI (30 min). The attachment of TA‐AF647 to the alginate‐TA backbone at a liquid/liquid and a solid/liquid interface was determined and compared. Here, TA‐647 was printed within 5% w/w PEG ink in 1% w/w alginate‐TA, which was subsequently photocrosslinked to assess the liquid/liquid interface. To investigate the solid/liquid interface, the first 5% w/w of PEG was printed in 1% w/w alginate‐TA, photocrosslinked, and perfused with 5% w/w PEG containing TA‐647 with and without crosslinker. Subsequent to crosslinking, all created channels were flushed with PBS and the samples were kept in PBS for 24 h to reduce background staining. The attachment of TA‐647 was confirmed using confocal fluorescence imaging (*N* = 3).

### Numerical Simulation

The shear stresses within a 260 µm (25 gauge) diameter blunt nozzle were modeled using COMSOL Multiphysics 5.6 software. The nozzle walls were selected to be solid boundaries with no slip. Input parameters were ink density and ink viscosity (Table S1, Supporting Information). As the used low‐viscosity ink reached a viscosity plateau already at low shear rates (10 s^−1^), the viscosity of this ink was set to be constant for all shear rates to 3.34 mPa∙s with a density of 1.032 g cm^−3^. For alginate (density 1.020 g cm^−3^), the ink viscosity was implemented utilizing the Carreau model (Equation (4)) with *µ_0_* being the zero shear rate and *µ_∞_* being the infinite shear rate viscosity and fitting parameters *λ* and *n*.

(4) $$\mu =\mu_{\infty }+\left(\mu_{0}-\mu_{\infty }\right)*{\left(1+{\left(\dot{\gamma }*\lambda \right)}^{2}\right)}^{\frac{n-1}{2}}$$

Input parameters were based on experimental rheological data. The shear thinning properties of 25% pluronic were modeled based on parameters reported by Paxton et al. ^[^ ^44^ ^]^ using the Power–Law model (Equation (5)) with *𝜼* being the viscosity, $\dot{\gamma }$ the shear rate, and *K* and *n* shear thinning coefficients.

(5) $$\eta =K\cdot {\dot{\gamma }}^{n-1}$$

The velocity, shear rate, and shear stress within the nozzle were selected as output parameters in either 2D or 3D models.

### Statistics

All graphs were created using OriginPro. Curve fitting and statistical analysis (one‐way ANOVA) were performed using OriginPro software. Schematics were created using Adobe Illustrator and BioRender.com. Adobe Photoshop was utilized for false coloring.

## Conflict of Interest

The authors declare no conflict of interest.

## Supporting information

Supporting Information

Supplemental Video 1

Supplemental Video 2

Supplemental Video 3

Supplemental Video 4

Supplemental Video 5

## Data Availability

The data that support the findings of this study are available from the corresponding author upon reasonable request.
