# Supplementary material for: Aqueous Two‐Phase Enabled Low Viscosity 3D (LoV3D) Bioprinting of Living Matter
Source: Adv Sci (Weinh). 2022 Dec 30;10(8):2204609. doi: 10.1002/advs.202204609 (PMC10015849; doi:10.1002/advs.202204609)
Supplement: Supplementary file 1 — Supporting Information [file ADVS-10-2204609-s001.pdf]

# Supporting Information

## Aqueous Two-Phase Enabled Low Viscosity 3D (LoV3D) Bioprinting of Living Matter

*Malin Becker*, Melvin Gurian, Maik Schot, Jeroen Leijten**

### Supplementary Methods

*Material synthesis:* Dextran-TA was synthesized via an adapted protocol from Jin *et al.*[1] Briefly, Dextran 40 kDa (5 g) and LiCl (4 g) were dissolved in anhydrous DMF (200 mL) under nitrogen atmosphere at 95 °C. After complete dissolution, the mixture was cooled to 0.5°C and p-nitro-phenyl chloroformate (PNC, 2 g) and pyridine (2 mL)were added. The mixture was let to react for 1h to form Dex-PNC, which was subsequently precipitated in ice cold ethanol. After filtration and washing with ethanol and diethyl ether, the product was dried under vacuum and stored in at 4 °C until further use. Dex-PNC (5 g) was dissolved in anhydrous DMF (100 mL) under nitrogen atmosphere. Tyramine was added in a 2:1 molar ratio to the attached PNC and let to react for 1h. Subsequently, the product was precipitated in ice cold ethanol. After filtration and washing with ethanol and diethyl ether the product was dried under vacuum. Subsequently, the product was dissolved in MilliQ water and dialyzed for 3 days against MilliQ using a 3.5 kDa MWCO membrane. The dialyzed solution was then lyophilized.

*$^{1}$H-NMR analysis:* Lyophilized polymer was dissolved in DMSO-$d_6$. For quantification of tyramine coupling, integrals of the peaks correlated with the dextran backbone (4.2-5.5, 4H) were compared to those of the phenyl of PNC (7.4-7.65 and 8.2-8.45, 2x2H) or tyramine moieties (6.6-6.75 and 6.9-7.07, 2x2H). The final polymer contained 4.4 tyramine moieties per 100 repetitive monosaccharide units.

*Granular bath preparation:* 2 % w/w gelatin (Bloom 300) and 11.23 % w/w PEG (35k) were dissolved in PBS. Under continuous stirring at 4 °C gelatin solution was added to PEG solution and cooled down for four hours. The gelatin granules were separated from the PEG solution via centrifugation, which were then washed three times using ice cold PBS. Subsequently particles were extruded through a 100 µm wide nozzle to obtain a more uniform size distribution. For use as an embedding bath, particles were incubated with 0.05% $H_2O_2$ overnight and PBS was removed to obtain a compact slurry.

*Angle analysis:* To investigate the influence of the initial angle to the printing angle, parallel lines were printed with 5% PEG in MilliQ water into 1% Alginate in MilliQ water. Then a drive through motion of a central line was performed at different angles between middle line and initial parallel lines. The deviation between initial and final angle (drag angle) was measured with initial angles of 30°, 45°, 60°, 75°, and 90° (N=3).

**Supplementary Figures**

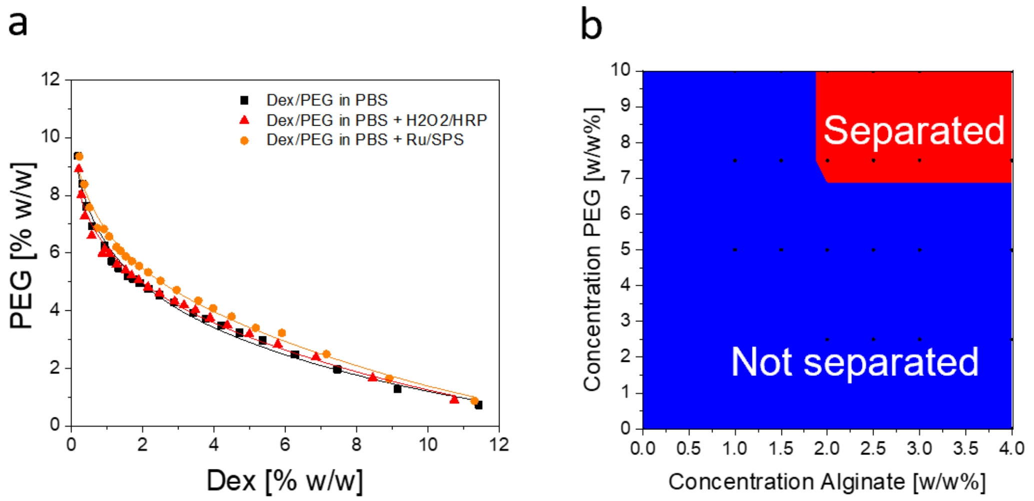


**Figure S1.** Binodal curve for selected polymer systems. a) Influence of added crosslinking agents to the Dextran/PEG binodal curve established including 1 mM Ru and 10 mM SPS as well as 0.05% $H_2O_2$ and 1 U $mL^{-1}$ HRP revealed that system stability was not adversely affected by the presence of crosslinking agents. b) Binodal curve of Alginate/PEG system due to the high viscosity of higher percentage of alginate solutions the phase separation was not confirmed by cloud point titration but via centrifugation of several concentration combinations. Occurrence of two phases was noted and is depicted for concentrations in red, while blue depicts concentrations where no separation was observed.

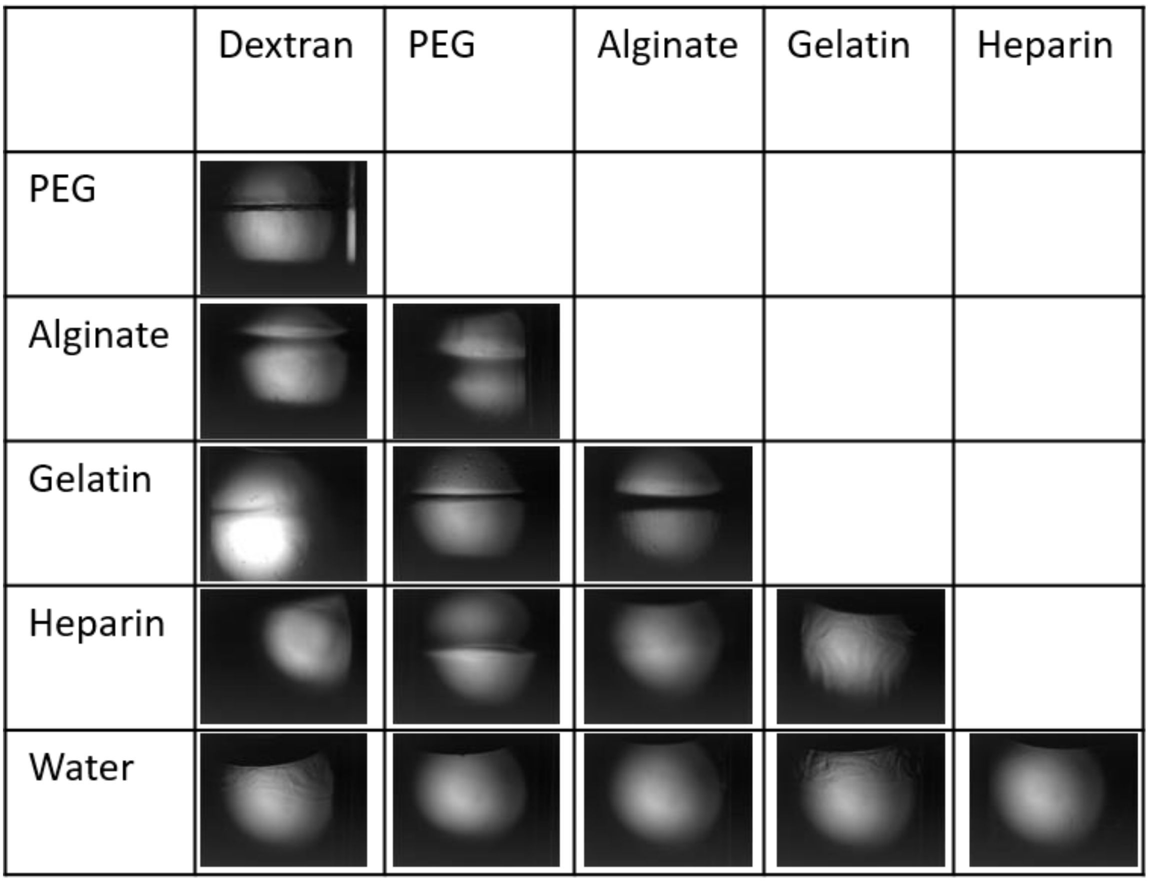


**Figure S2.** Interfacial stability of polymer solutions commonly utilized for tissue engineering applications. Representative images of interfaces after 60 min of various polymer solution combinations. Dextran. PEG, gelatin, and heparin solutions contained 10% w/w, and alginate contained 2% w/w polymer in MilliQ water.

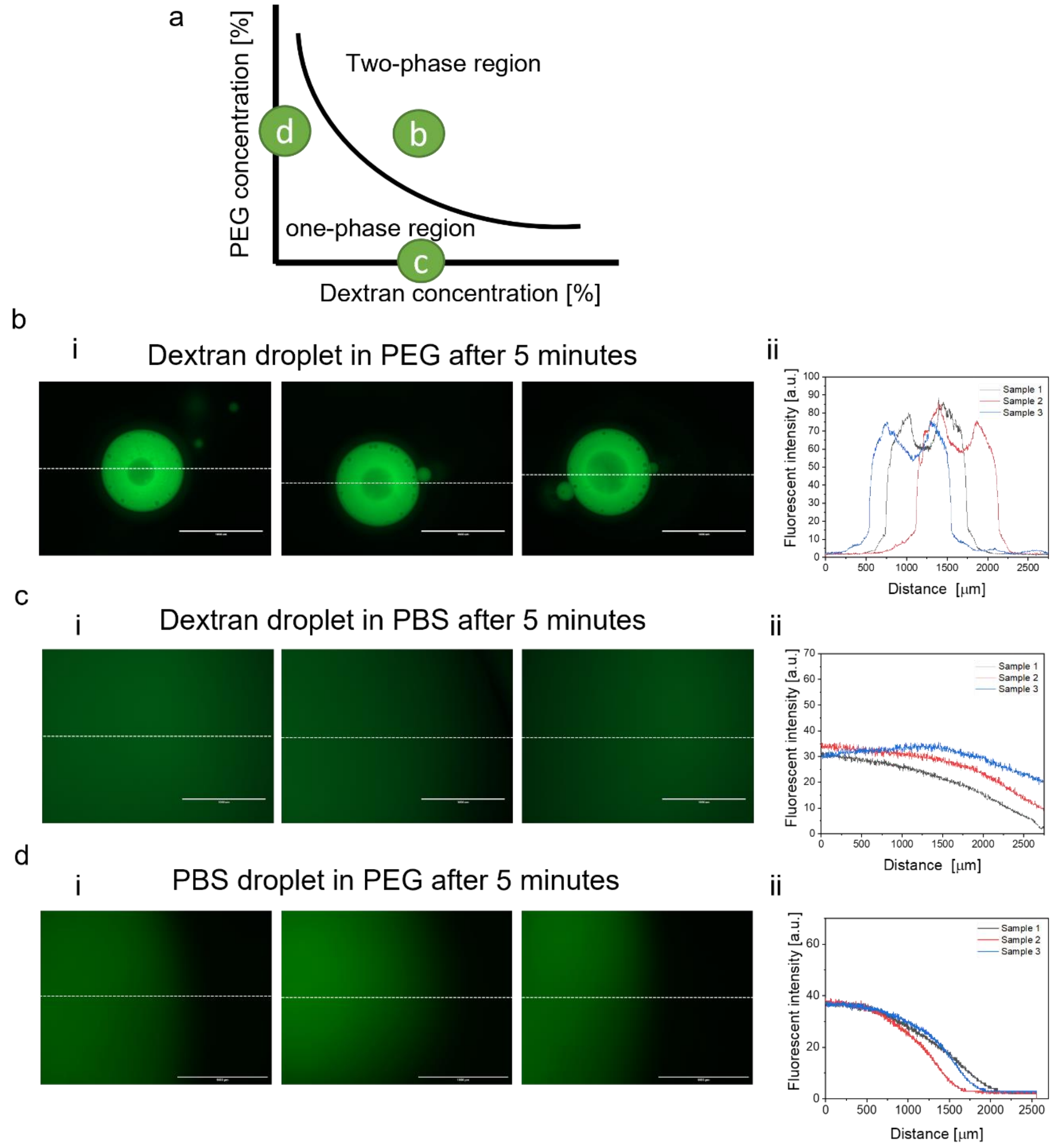


**Figure S3.** Assessment of interface stability of an ATPS system within the two-phase and one-phase region. a) Schematic depiction of the binodal curve of a dextran and PEG system with indications of the regimes utilized in b,c, and d. Representative fluorescent micrographs of b) 1μL 5% dextran containing dextran-FITC within a 11.23% W/W PEG bath or c) within a PBS bath as well as d) 1 μL PBS containing fluorescein sodium salt in a 11.23% PEG bath after 5 minutes with i) fluorescent images of the samples after 5 minutes and ii) their respective intensity profiles. (N=3). Scale bars: (b,i), (c,i), (d,i) 1mm.

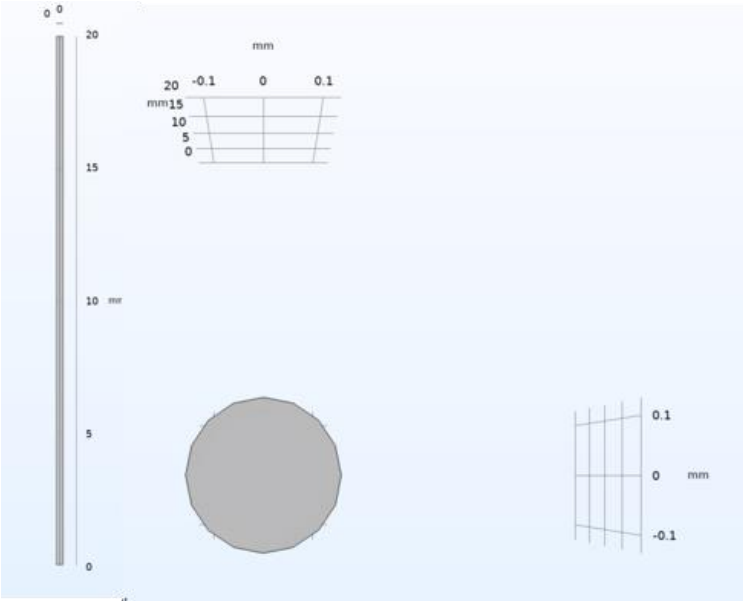

**Figure S4.** 3D nozzle model utilized for the COMSOL modelling with a diameter of 260 µm and a length of 2000 µm. Slice plots of the shear stresses within the nozzle were taken at 1000 µm nozzle length.

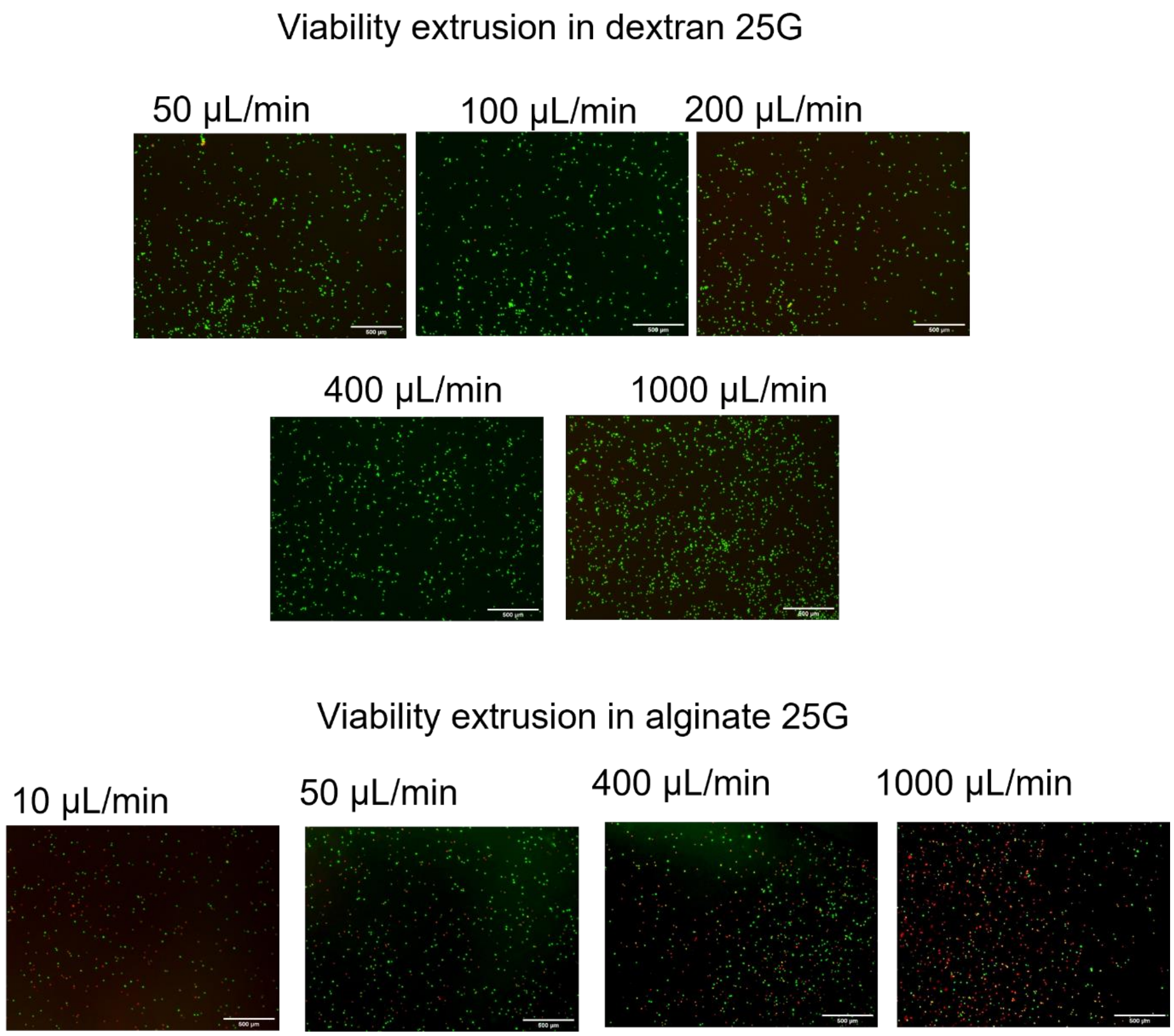


**Figure S5.** Representative live dead images utilized to assess the viability of 3T3 cells four hours after extrusion. Cells were extruded through a 25G nozzle in the ink and at the flow rate indicated above the images. Scale bars: 500 µm.

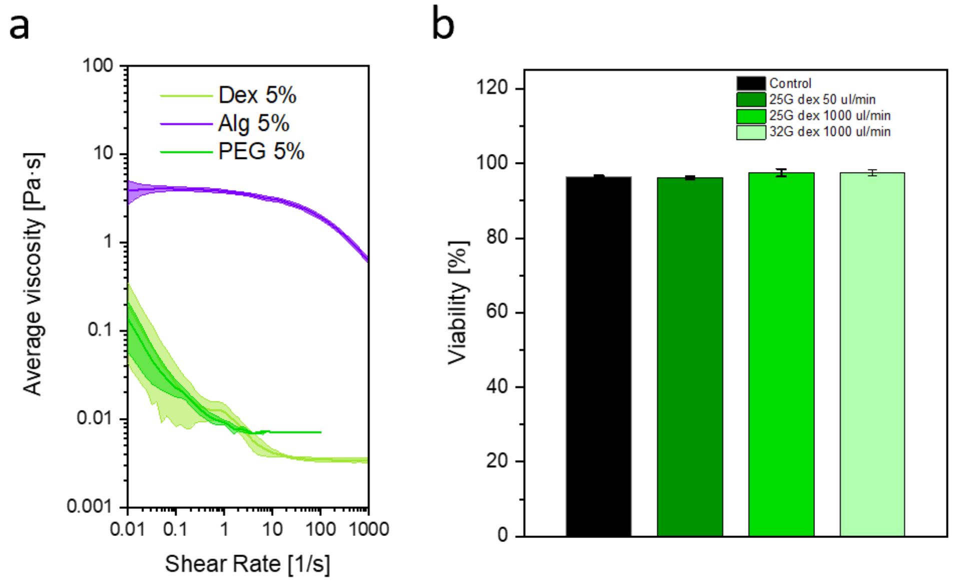


**Figure S6.** Viscosity and cell viability of employed ink solutions. a) viscosity under various shear rates were determined for 5% dextran, 5% PEG low viscous inks of which the printing is enabled by LoV3D printing as well as alginate as a high viscosity control. The viscosity plateau reached at 10 $s^{-1}$ for dextran and PEG was utilized for shear stress modelling. b) Viability of 3T3 cells within 5% dextran after extrusion through a 25G and 32G nozzle. The viability was not reduced compared to a non-extruded control condition.

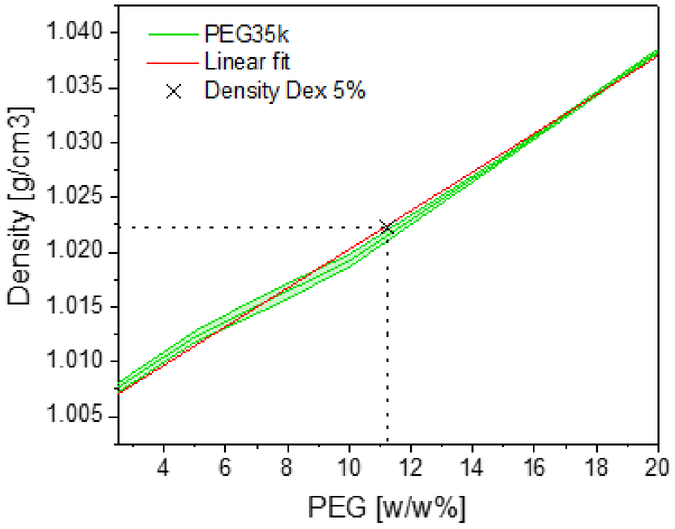


**Figure S7.** Density of PEG solutions with varying concentrations (green) and concentration selected to match the density of the employed dextran bath (cross). The density was assessed

with a pycnometer and the PEG% matching the density of the 5% dextran solution was found to be 11.23% w/w.

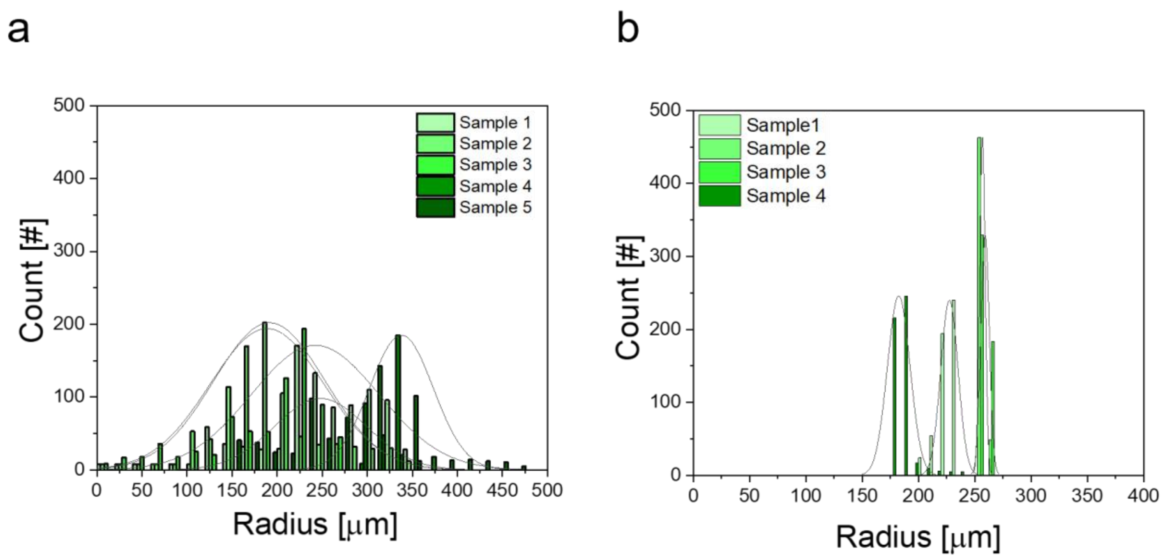


**Figure S8.** Histogram depicting the distribution of radii measured from strands printed withing **a)** granular embedding baths or **b)** low viscous aqueous baths using the LoV3D method.

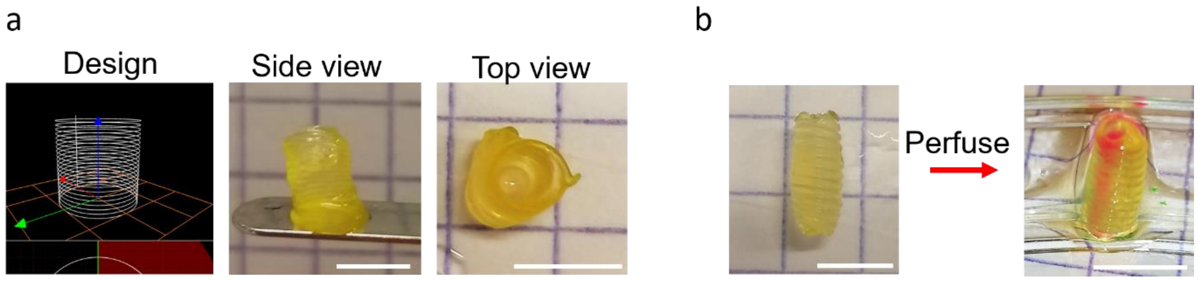


**Figure S9.** 3D printed structures using 5% Dex-TA as an ink that was enzymatically crosslinked after printing within a 11.23% PEG bath. **a)** Initial design as well as top and side view images from a 3 mm diameter tube after removal from the embedding bath. **b)** Images of a printed tube before and after perfusion with an aqueous ink containing Rhodamine B. Scale bars: (a), (b) 5 mm.

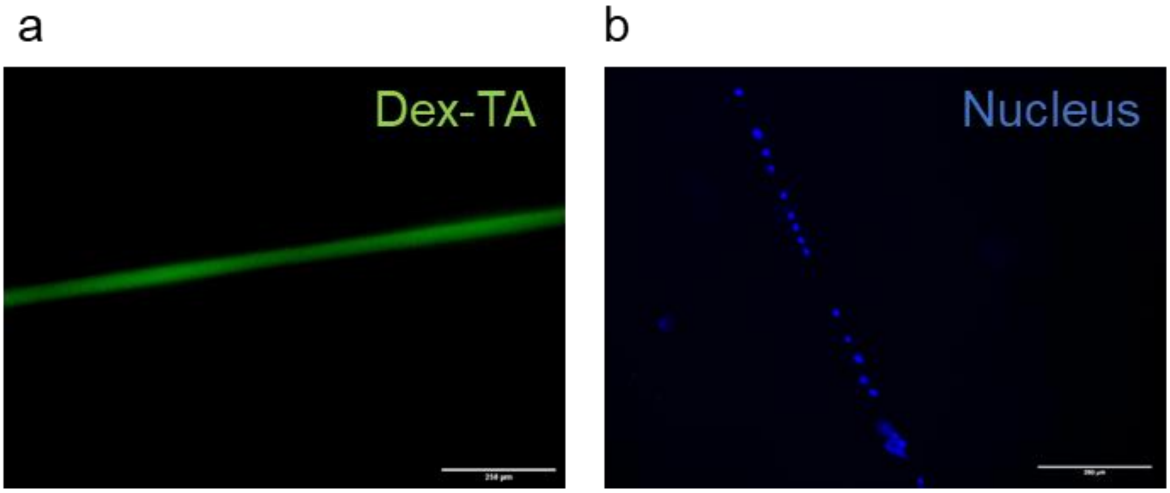


**Figure S10.** Printing at single cell resolution through a 260 µm diameter nozzle. a) Confocal image of a Dex-TA hydrogel thread printed at high speed. b) Confocal image of 3T3 cells with stained nucleus (blue) indicating single cell resolution. Scale bars: (a,b) 250 µm.

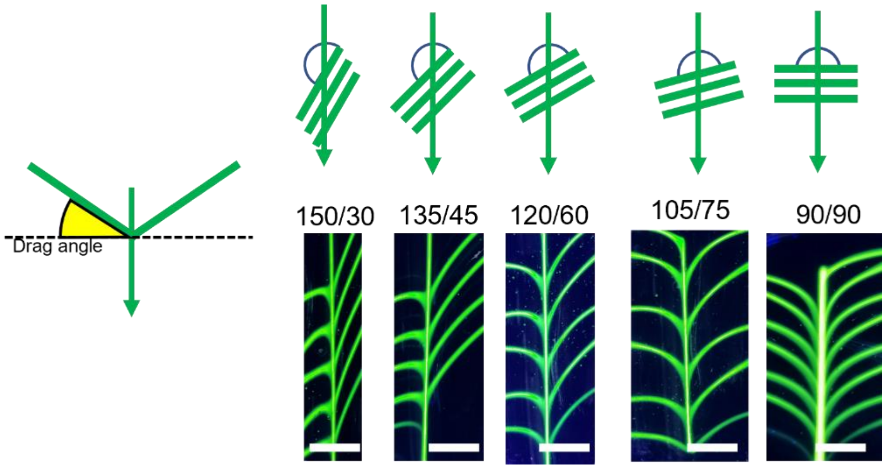


**Figure S11.** Set-up of drag angle analysis. a) The drag angle is defined as angle deviation between the initial (theoretical) and the final angle (left). b) The drag angle was assessed for initial angles ranging from 30-120°. Representative fluorescence microphotographs are shown for each initial angle. Scale bars: 5 mm.

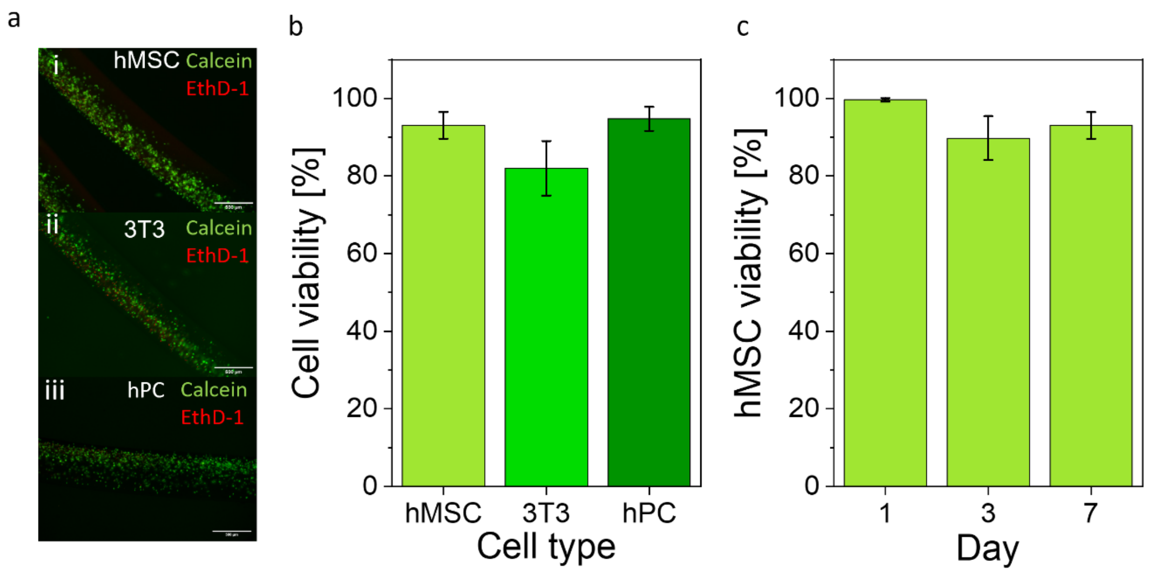


**Figure S12.** Viability analysis of cells printing within a 5% Dex-TA bioink containing 1 U mL$^{-1}$HRP in a 11.23% PEG bath containing 0.05% $H_2O_2$. a) representative live dead images at day 7. b) Cell viability of printed hMSCs, 3T3s and hPCs after seven days of culture (N=10). c) Viability of hMSCs within the Dex-TA bioink at different time points (N=10). Scale bars: (a) 500 μm.

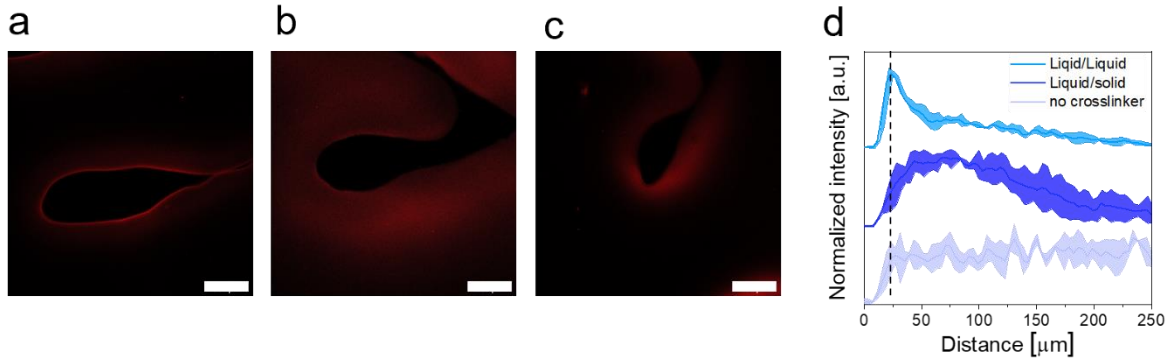


**Figure S13.** Confocal images of atto-Tyramide spatial distribution for a) liquid/liquid and solid/liquid interface b) with and c) without crosslinker. d) Intensity profiles of the channel interface (interface indicated by dotted line). The dye was covalently attached for all conditions containing crosslinker solutions, however, showed a more localized functionalization for the liquid/liquid interface. The presence of no crosslinker resulted in a stable background, indicating that all spatial patterns are indeed attributed to covalent dye attachment in the presence of crosslinkers. Scale bars: 250 μm.

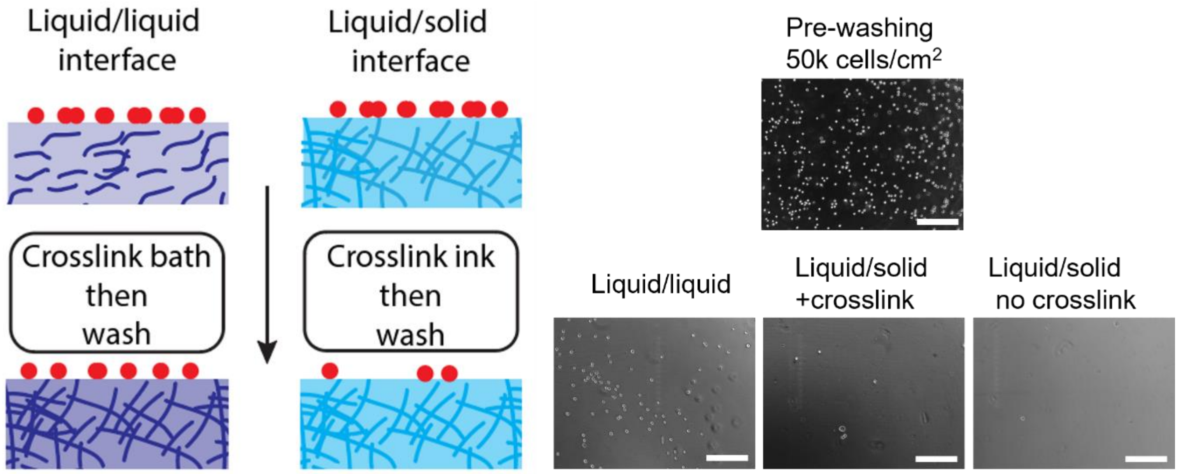


**Figure S14.** Cell attachment at liquid/liquid and liquid/solid interfaces. a) Schematic depicting experimental design. Specifically, ink containing cells in the presence of absence of a crosslinker were placed onto a hydrogel (precursor) and let to react with a liquid/liquid or liquid/solid interface. b) Microphotographs of resulting samples before and after washing for all conditions. Scale bars: 250 µm.

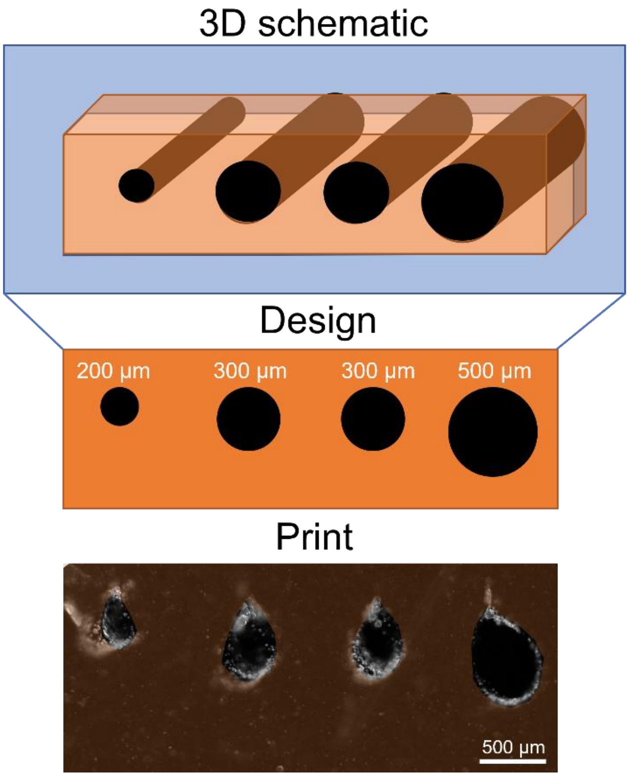


**Figure S15.** Schematic depiction of the initial design and microphotograph of actual printing outcome of a sacrificial PEG ink (5% w/w PEG, $10^7$ cells mL$^{-1}$(3T3 cells) with 0.01 mg mL$^{-1}$ collagen type I) with channel diameters of ~200, ~300, ~300 and ~500 µm (left to right) within a photocrosslinked alginate-TA bulk. Scale bar: 500 µm.

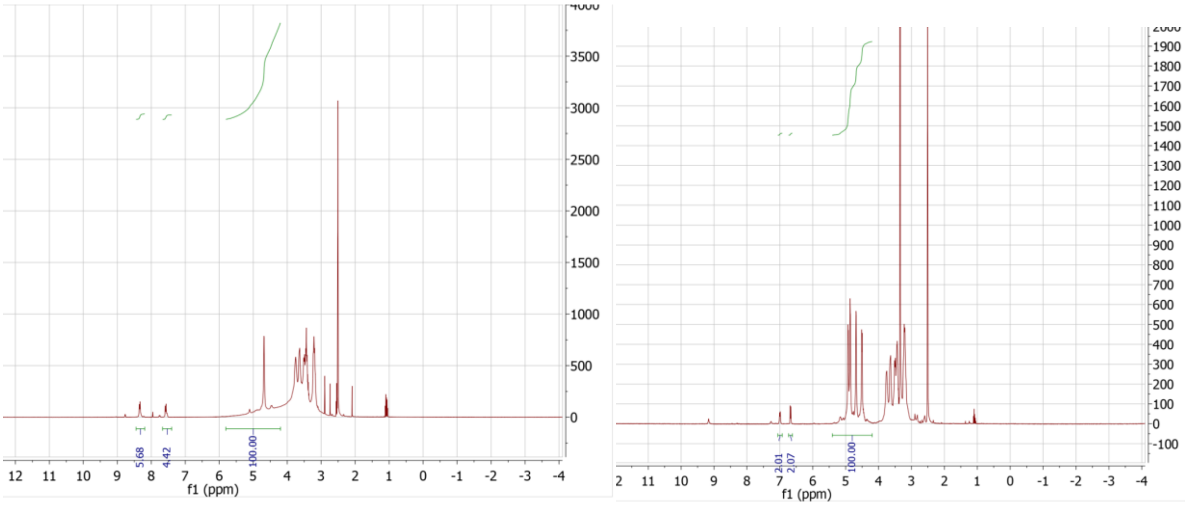


**Figure S16.** H-NMR spectrum of (left) Dextran-PNC and (right) Dextran-Tyramine confirming successful functionalization with a degree of substitution of 4%.

**Table S1**. Viscosity and respective product names utilized to create a viscosity range indicator.

| Product name | Viscosity [mPas] | Supplier | Catalog Number |
|---|---|---|---|
| Alginic acid sodium salt, **very low viscosity** | 4-12 | Thermo Fischer Scientific | A18565.36 |
| Alginic acid sodium salt, **low viscosity** | 30-90<br>4-12 | Thermo Fischer Scientific<br>Sigma | B25266.36<br>A11122 |
| Alginic acid sodium salt, **medium viscosity** | ≥ 2000<br>600-900 | Sigma<br>Fischer Scientific | A2033<br>11464751 |
| Alginic acid sodium salt, **high viscosity** | 1000-1500<br>14000 | Fischer Scientific<br>Sigma | J61887.A1<br>A7128 |

**Table S2.** Material parameters selected as input for COMSOL simulation of shear rates and shear stresses within a nozzle during printing. Alginate was fitted with the Carreau model with $\mu_0$ being the zero shear rate and $\mu_\infty$ being the infinite shear rate viscosity and fitting parameters $\lambda$ and $n$. Pluronic was fitted with the Powerlaw model with $\eta$ being the viscosity, $\dot{\gamma}$ the shear rate, and $K$ and $n$ shear thinning coefficients.

| Material | Parameter source | $\mu_0$ | $\mu_\infty$ | n | K | n | Density [kg m$^{-3}$] | λ | $R^2$ |
|---|---|---|---|---|---|---|---|---|---|
| Alginate 5% | Experimental | 4 | 0.0001 | | | | | | |
| | Carreau fit | | | 0.65743 | | | | 0.12623 | 0.9868 |
| Pluronic PF-127 25% | Literature[2] | | | | 406 | 0.127 | 1040 | | |

**Table S3.** Material parameters of solutions chosen for the sedimentation study.

| Dextran MW [kDa] | Concentration [% w/w] | Viscosity plateau [mPa·s] | Density [g cm$^{-3}$] | PEG MW [kDa] | Concentration [% w/w] | Density [g cm$^{-3}$] |
|---|---|---|---|---|---|---|
| 40 | 5 | 2.18 | 1.022 | 35 | 5 | 1.012 |
| 500 | 5 | 13.96 | 1.022 | 35 | 10 | 1.02 |
| 500 | 10 | 39.32 | 1.043 | 35 | 15 | 1.03 |
| 500 | 20 | 224.01 | 1.08[a)] | 35 | 20 | 1.04 |
| 500 | 30 | 1222.08 | 1.13[a)] | 35 | 50 | 1.09[a)] |

[a)] calculated from linear fit

**Table S4.** References and respective utilized data points for the literature study.

| Reference | Ink viscosity at max. reported shear rate [Pa·s] | Bath viscosity (steady) [Pa·s] | Nozzle diameter [µm] | Lowest diameter [µm] |
|---|---|---|---|---|
| Wu, 2011[3] | 1 at 100 $s^{-1}$ | 10000-12 at 10 $s^{-1}$ and 1 $s^{-1}$ | | |
| Spencer, 2019 [4] | 0.1 at 1000 $s^{-1}$ | | 160 | 120 |
| Noor, 2019 [5] | | | 160 | 110 |
| Montalbano, 2020 [6] | 0.14 at 1000 $s^{-1}$ | | 120 | 120 |
| Mendes, 2019[7] | | 100 at 0.01 $s^{-1}$ | 150 | 100 |
| Lindsay, 2019 [8] | 5 at 10 $s^{-1}$ | | 412 | 400 |
| Skylar-Scott, 2019 [9] | 10 at 10 $s^{-1}$ | | | |
| Lee, 2020 [10] | 0.162 at 50 $s^{-1}$ | | | |
| Jeon, 2019 [11] | | | 412<br>260<br>210 | 395<br>310<br>276 |
| Cidonio, 2019 [12] | ~1 at 100 $s^{-1}$ | | 250 | 280 |
| Choi, 2019 [13] | | | 210 | 300 |
| Luo [14] | 0.3 at 100 $s^{-1}$ | | | |
| Ning [15] | | | 210 | 200 |

**References**


[1] R. Jin, C. Hiemstra, Z. Zhong, J. Feijen, *Biomaterials* **2007**, 28, 2791.
[2] E. Reina-Romo, S. Mandal, P. Amorim, V. Bloemen, E. Ferraris, L. Geris, *Front Bioeng Biotechnol* **2021**, 9, 701778.
[3] W. Wu, A. DeConinck, J. A. Lewis, *Adv Mater* **2011**, 23, H178.
[4] A. R. Spencer, E. Shirzaei Sani, J. R. Soucy, C. C. Corbet, A. Primbetova, R. A. Koppes, N. Annabi, *ACS Appl Mater Interfaces* **2019**, 11, 30518.
[5] N. Noor, A. Shapira, R. Edri, I. Gal, L. Wertheim, T. Dvir, *Adv Sci (Weinh)* **2019**, 6, 1900344.
[6] G. Montalbano, G. Molino, S. Fiorilli, C. Vitale-Brovarone, *Journal of the European Ceramic Society* **2020**, 40, 3689.
[7] B. B. Mendes, M. Gomez-Florit, A. G. Hamilton, M. S. Detamore, R. M. A. Domingues, R. L. Reis, M. E. Gomes, *Biofabrication* **2019**, 12, 015012.
[8] C. D. Lindsay, J. G. Roth, B. L. LeSavage, S. C. Heilshorn, *Acta Biomater* **2019**, 95, 225.
[9] M. A. Skylar-Scott, S. G. M. Uzel, L. L. Nam, J. H. Ahrens, R. L. Truby, S. Damaraju, J. A. Lewis, *Sci Adv* **2019**, 5, eaaw2459.
[10] S. Lee, E. S. Sani, A. R. Spencer, Y. Guan, A. S. Weiss, N. Annabi, *Adv Mater* **2020**, 32, e2003915.
[11] O. Jeon, Y. B. Lee, H. Jeong, S. J. Lee, D. Wells, E. Alsberg, *Mater Horiz* **2019**, 6, 1625.
[12] G. Cidonio, M. Cooke, M. Glinka, J. I. Dawson, L. Grover, R. O. C. Oreffo, *Mater Today Bio* **2019**, 4, 100028.
[13] Y. J. Choi, Y. J. Jun, D. Y. Kim, H. G. Yi, S. H. Chae, J. Kang, J. Lee, G. Gao, J. S. Kong, J. Jang, W. K. Chung, J. W. Rhie, D. W. Cho, *Biomaterials* **2019**, 206, 160.
[14] G. Luo, Y. Yu, Y. Yuan, X. Chen, Z. Liu, T. Kong, *Adv Mater* **2019**, 31, e1904631.
[15] L. Ning, R. Mehta, C. Cao, A. Theus, M. Tomov, N. Zhu, E. R. Weeks, H. Bauser-Heaton, V. Serpooshan, *ACS Appl Mater Interfaces* **2020**, 12, 44563.
